# A GMCSF-Neuroantigen Tolerogenic Vaccine Elicits Systemic Lymphocytosis of CD4^+^ CD25^high^ FOXP3^+^ Regulatory T Cells in Myelin-Specific TCR Transgenic Mice Contingent Upon Low-Efficiency T Cell Antigen Receptor Recognition

**DOI:** 10.3389/fimmu.2018.03119

**Published:** 2019-01-10

**Authors:** Cody D. Moorman, Alan D. Curtis, Alexander G. Bastian, Sarah E. Elliott, Mark D. Mannie

**Affiliations:** Department of Microbiology and Immunology, Brody School of Medicine, East Carolina University, Greenville, NC, United States

**Keywords:** FOXP3, Tregs, GM-CSF, neuroantigen, EAE, tolerance, multiple sclerosis

## Abstract

Previous studies showed that single-chain fusion proteins comprised of GM-CSF and major encephalitogenic peptides of myelin, when injected subcutaneously in saline, were potent tolerogenic vaccines that suppressed experimental autoimmune encephalomyelitis (EAE) in rats and mice. These tolerogenic vaccines exhibited dominant suppressive activity in inflammatory environments even when emulsified in Complete Freund's Adjuvant (CFA). The current study provides evidence that the mechanism of tolerance was dependent upon vaccine-induced regulatory CD25^+^ T cells (Tregs), because treatment of mice with the Treg-depleting anti-CD25 mAb PC61 reversed tolerance. To assess tolerogenic mechanisms, we focused on 2D2-FIG mice, which have a transgenic T cell repertoire that recognizes myelin oligodendrocyte glycoprotein peptide MOG35-55 as a low-affinity ligand and the neurofilament medium peptide NFM13-37 as a high-affinity ligand. Notably, a single subcutaneous vaccination of GMCSF-MOG in saline elicited a major population of FOXP3^+^ Tregs that appeared within 3 days, was sustained over several weeks, expressed canonical Treg markers, and was present systemically at high frequencies in the blood, spleen, and lymph nodes. Subcutaneous and intravenous injections of GMCSF-MOG were equally effective for induction of FOXP3^+^ Tregs. Repeated booster vaccinations with GMCSF-MOG elicited FOXP3 expression in over 40% of all circulating T cells. Covalent linkage of GM-CSF with MOG35-55 was required for Treg induction whereas vaccination with GM-CSF and MOG35-55 as separate molecules lacked Treg-inductive activity. GMCSF-MOG elicited high levels of Tregs even when administered in immunogenic adjuvants such as CFA or Alum. Conversely, incorporation of GM-CSF and MOG35-55 as separate molecules in CFA did not support Treg induction. The ability of the vaccine to induce Tregs was dependent upon the efficiency of T cell antigen recognition, because vaccination of 2D2-FIG or OTII-FIG mice with the high-affinity ligands GMCSF-NFM or GMCSF-OVA (Ovalbumin323-339), respectively, did not elicit Tregs. Comparison of 2D2-FIG and 2D2-FIG-*Rag1*^−/−^ strains revealed that GMCSF-MOG may predominantly drive Treg expansion because the kinetics of vaccine-induced Treg emergence was a function of pre-existing Treg levels. In conclusion, these findings indicate that the antigenic domain of the GMCSF-NAg tolerogenic vaccine is critical in setting the balance between regulatory and conventional T cell responses in both quiescent and inflammatory environments.

## Introduction

Multiple Sclerosis (MS) is an inflammatory demyelinating disease of the central nervous system (CNS) marked by periodic focal attacks on white and gray matter myelin accompanied by significant diffuse myelin and axonal injury and atrophy (1–7). MS often begins as an overt relapsing-remitting disease accompanied by an insidious, clinically-subvert progression of disability. After 10–25 years, the disease often transitions from a succession of punctate attacks to a secondary progressive phase marked by incessant white and gray matter degeneration and increasing levels of physical and cognitive impairment (8, 9). MS is widely considered to be an autoimmune disease of the CNS driven by molecular mimicry against environmental antigens complemented by genetic influences that drive autoimmunity and impair the homeostatic regulatory responses needed to maintain tolerance in CNS tissues (10–12). Several studies have provided evidence that MS is associated with deficient function of regulatory T cells, including the canonical CD25^high^ FOXP3^+^ regulatory T cells (Tregs) (13–15). Current therapies for MS however are not designed to restore a homeostatic balance needed for adaptive tolerance and disease resolution. Instead, first-line immunomodulatory drugs lack disease-specific activity and thereby exhibit modest efficacy and do not alter the long-term disease trajectory in most MS patients (16). Second-line broad-spectrum, immunosuppressive drugs block both pathogenic and adaptive immunity and may result in an immunocompromised state, opportunistic infection, and/or cancer (17–19). Tolerogenic vaccines are qualitatively distinct and are designed to restore myelin-specific Treg responses and specifically repair a major defect underlying MS susceptibility. Tolerogenic vaccines for MS that have been tested in pre-clinical models of EAE are based on diverse technical platforms that include naked synthetic peptides, DNA vectors, antigen-expressing dendritic cell APC/leukocytes, engineered viral vectors, antigen-bearing vesicles/nanoparticles, among several others (20–26). Many of these vaccine platforms however lack robust therapeutic efficacy (27). Ideally, tolerogenic vaccines should exhibit prophylactic and therapeutic activity, attenuate the pathogenic T conventional cell (Tcon) repertoire, and enhance the Treg repertoire even within the confines of proinflammatory environments (28).

Fusion proteins containing GM-CSF as the N-terminal domain and a dominant encephalitogenic neuroantigen (NAg) as the C-terminal domain represent an emerging vaccine platform that may fulfill these criteria. GMCSF-NAg tolerogenic vaccines were effective in three different EAE models including monophasic EAE in Lewis rats, relapsing-remitting EAE in SJL mice, and chronic EAE in C57BL/6 mice (28–32). Recombinant proteins comprised of rat GM-CSF fused to the Myelin Basic Protein (MBP) 69–87 peptide, murine GM-CSF fused to the Proteolipid Protein (PLP)139–151 peptide, and murine GM-CSF fused to the MOG35-55 peptide were used in the respective EAE models. In each system, GMCSF-NAg was an effective prophylactic that prevented the subsequent induction of EAE. GMCSF-NAg was also a therapeutic intervention that elicited remission when administered after the onset of severe paralytic EAE (29–32). The tolerogenic mechanism of GMCSF-MOG was unique because the fusion protein did not require a quiescent environment to inhibit EAE (29). Rather, GMCSF-MOG was effective when administered adjacent to or within a MOG35-55/CFA encephalitogenic emulsion even when MOG35-55 was at a 75:1 molar excess compared to GMCSF-MOG. This observation negates the dogma that tolerogenic vaccines require a quiescent non-inflammatory environment for the efficient induction of tolerance. The relevant target of GMCSF-NAg *in vivo* is thought to be myeloid APC, because *in vitro* analyses revealed that GMCSF-NAg fusion proteins targeted NAg for enhanced antigen presentation by myeloid APC *in vitro*. For example, a GMCSF-NAg fusion protein that included the 69–87 epitope of MBP engendered an approximate 1,000-fold enhancement of antigenic potency by a mechanism that was blocked by free GM-CSF and that depended on physical linkage of the GM-CSF and NAg domains (32). Interleukin-4 (IL4)-NAg and macrophage colony stimulating factor (MCSF)-NAg fusion proteins targeted NAg for enhanced presentation by B cells and macrophages, respectively. But IL4-NAg lacked tolerogenic activity (33), and MCSF-NAg did not approximate the tolerogenic activity of GMCSF-NAg (32). These findings suggested that GMCSF-NAg fusion proteins have the core activities needed for tolerogenic vaccination.

The use of GM-CSF as a tolerogenic fusion partner is intriguing because GM-CSF has classically been considered a proinflammatory cytokine, and GM-CSF has been successfully used as an immunogenic domain in GMCSF-antigen fusion vaccines in models of cancer and infectious disease (34–42). Nonetheless, GM-CSF is also known to elicit differentiation of regulatory DC and myeloid-derived suppressor cells that in turn elicit regulatory T cells to inhibit autoimmune disease (43–45). Administration of GM-CSF inhibits autoimmune disease in several pre-clinical disease models, including experimental myasthenia gravis, autoimmune diabetes, and experimental autoimmune thyroiditis (46–55). Conversely, deficiency of GM-CSF is associated with susceptibility to autoimmune diabetes and systemic lupus erythematosus (56, 57). The activity spectrum of GM-CSF is therefore complex and contradicts simple perspectives as a pro-inflammatory or anti-inflammatory cytokine. One possibility is that GM-CSF amplifies immunogenic or tolerogenic activity of associated antigens based on the intrinsic T cell antigen receptor recognition events that are dominant in a particular locale. If so, then GMCSF-NAg fusion proteins would amplify the intrinsic immunogenic or tolerogenic activity of the covalently-tethered NAg domain.

To address this hypothesis, the current study focused on 2D2-FIG strain of MOG-specific mice that have a transgenic T cell repertoire specific for the low affinity ligand MOG35-55. This study revealed that subcutaneous (SC) administration of GMCSF-MOG in saline elicited a major population of FOXP3^+^ Tregs comprising ~20–40% of all circulating T cells within ~3–4 days. The GMCSF-MOG vaccine also imposed a desensitized phenotype upon the 2D2 T cell repertoire, as shown by reduced circulating T cell numbers, down-regulated CD3/TCR expression on a per cell basis, and expanded percentages of CD4^(−)^ 2D2 T cells. In contrast, GMCSF-based fusion proteins incorporating highly agonistic antigens NFM13-37 or OVA323-337 lacked robust Treg-induction activity in 2D2-FIG and OTII-FIG models, respectively. Thus, the antigen-targeting and adjuvant activities of the GM-CSF domain of GMCSF-antigen fusion proteins may simply amplify the intrinsic antigenic activity and efficiency of TCR-antigen recognition events to set the balance of Treg/Tcon responses. These data indicate that the vaccine-targeted presentation of the low-affinity MOG35-55/I-A^b^ by myeloid APC is a key parameter for induction of a predominant Treg response and establishment of CNS-specific tolerance. Because CNS myelin peptides are uniformly subject to self-tolerance and exhibit weak, inefficient interactions with the T cell repertoire, GMCSF-NAg fusion proteins may have wide applicability as tolerogenic vaccines in CNS-targeted autoimmune disease. The extrapolation is that most self-proteins, when incorporated as GMCSF-antigen fusion proteins, would drive dominant Treg responses to elicit immunological tolerance.

## Materials and Methods

### Mice

C57BL/6J (000664), B6.Cg-*Foxp3*^*tm*2*Tch*^/J (FIG Foxp3-IRES-GFP 006772), B6.129S7-*Rag1*^*tm*1*Mom*^/J (*Rag1*^−/−^ 002216), C57BL/6-Tg(Tcra2D2,Tcrb2D2)1Kuch/J (2D2 MOG35-55-specific TCR transgenic 006912), and B6.Cg-Tg(TcraTcrb)425Cbn/J (OTII OVA323-339-specific TCR transgenic 004194) mouse strains were obtained from the Jackson Laboratory (Bar Harbor, ME) and were housed and bred in the Department of Comparative Medicine at East Carolina University Brody School of Medicine. 2D2-FIG, 2D2-FIG-*Rag1*^−/−^, and OTII-FIG mice were obtained through intercross breeding. Animal care and use was performed in accordance with approved animal use protocols and guidelines of the East Carolina University Institutional Animal Care and Use Committee.

### Reagents and Recombinant Proteins

Synthetic peptides MOG35-55 (MEVGWYRSPFSRVVHLYRNGK), NFM13-37 (RRVTETRSSFSRVSGSPSSGFRSQS), and OVA323-339 (ISQAVHAAHAEINEAGR) were obtained from Genscript (Piscataway, NJ). Derivation, expression, purification, and bioassay of the murine GM-CSF and GMCSF-MOG fusion proteins were described in previous studies (30, 31). These fusion proteins as well as GMCSF-OVA and GMCSF-NFM were comprised of the murine GM-CSF cytokine as the N-terminal domain, the amino acid sequence comprising the relevant antigenic peptide domain, and an 8-histidine C-terminus. GM-CSF contained the 8-histidine tag C-terminus but did not contain an antigenic peptide domain. GMCSF-MOG, GMCSF-NFM, and GMCSF-OVA contained MOG35-55, NFM13-37, and OVA323-339 peptides, respectively. These fusion proteins did not contain linkers in the GM-CSF/antigenic peptide/8-histidine-tag junctions. These recombinant proteins were isolated from stably-transfected human embryonic kidney (HEK) cells or from Chinese hamster ovary (CHO) cells. Expression supernatants were concentrated on YM10 ultrafiltration membranes and were directly applied to Ni-NTA Agarose columns (Qiagen, Chatsworth, CA) followed by extensive washing of the resin bed (50 mM NaH_2_PO_4_, 500 mM NaCl, with 10, 20, or 60 mM imidazole, pH 8.0). Recombinant proteins were eluted with 250 mM imidazole (pH 8.0) and were concentrated and diafiltrated in Amicon Ultra-15 centrifugal filter devices (EMD Millipore, Billerica, MA). Protein quantity was assessed by absorbance at 280 nm, and purity was assessed by SDS-PAGE.

### Generation, Purification, and Administration of Monoclonal Antibodies (mAbs)

Hybridomas secreting the PC61-5.3 mAb or a rat IgG1 isotype control were described previously (58). Hybridoma cells were cultured in supplemented DMEM in C2011 hollow fiber cartridges (FiberCell Systems, Inc., Frederick, MD). Hybridoma supernatants were clarified at 7,200 x g, precipitated with 50% ammonium sulfate, and dissolved in PBS. MAb preparations were purified on protein G agarose columns. Antibody was eluted with 200 mM glycine at pH 3.0 and immediately neutralized by 1M Tris buffer of pH 9.0. The purity of the antibody was verified by SDS-PAGE.

### Flow Cytometric Analyses of Lymphocytes, Splenocytes, and Peripheral Blood Mononuclear Cells (PBMC)

Blood was collected from the submandibular vein into 200 μl of sodium citrate (130 mM). Inguinal lymph nodes and spleen were dissected from mice and placed into 10 ml of HBSS. Dissected lymph nodes and spleen were pressed through a wire mesh screen and a 70 μm cell strainer (Corning, NY) to obtain single-cell suspensions. Cells were washed in 3 ml HBSS with 2% heat-inactivated FBS and stained with designated cocktails of fluorochrome-conjugated antibodies for 1 h at 4°C in the dark. After staining whole blood, erythrocytes (RBC) were lysed with 1:10 HBSS for 20 s at 4°C followed by addition of 2X PBS. Alternatively, RBCs were lysed by incubating samples for 10 min on ice with 3 ml of ammonium chloride lysis buffer (150 mM NH_4_Cl, 10 mM NaHCO_3_, 1.2 mM EDTA- pH 7.2). Lysis was repeated when necessary. Samples were then washed 1 time with HBSS/2% FBS and were analyzed on a Becton-Dickson LSRII flow cytometer (San Jose, CA) with FlowJo software (Ashland, OR). In designated experiments, reference “counting” beads were added to samples immediately before flow cytometric analysis (AccuCount blank particles or FITC-, PE-, or APC-conjugated EasyComp fluorescent particles 3.0–3.4 μm, Spherotech, Lake Forest, IL). The use of reference beads enabled comparisons of absolute cell numbers among different samples. For intercellular staining of FOXP3 and Ki67, blood was collected, and RBCs were lysed as previously described. PBMC were fixed for 10 min using 2% paraformaldehyde (PFA) and were fixed/permeabilized with 1 mL of ice cold 100% methanol for 30 min. Cells were then stained with antibody cocktails against both surface markers and intercellular targets for 30 min at room temp. Cells were extensively washed between PFA, methanol, and staining treatments using PBS + 2% FBS. Fluorochrome-conjugated mAbs were obtained from BioLegend and included CD3-BV421, PE/Dazzle 594, or PE (17A2 or 145-2C11), CD4-BV785, PE, or APC (GK1.5), CD25-BV421 (PC61), CD44-BV421 (IM7), CD45-BV785 (30-F11), CD62L-APC (MEL-14), TCR-Vα3.2-PE (RR3-16), TCR-Vβ11-PE or AF647 (KT11), TCR-Vβ5.1,5.2-PE (MR9-4), TCR-Vα2-APC (B20.1), Neuropilin-PE (3E12), and LAP-PE (TW7-16B4). Comparisons among three or more groups were analyzed by use of ANOVA, which was interpreted with a Holm-Sidak multiple comparisons test. Pairwise comparisons were analyzed by two-tailed *t*-tests for data that passed Normality (Shapiro-Wilk) and Equal Variance (Brown-Forsythe) tests. A *P*-value < 0.05 was considered significant. Error bars represent standard error of the mean (SE) unless designated otherwise.

### Measurement of GM-CSF Activity and Antigen-Specific T Cell Responses

To measure antigen-specific proliferation, 2D2 or OTII T cells (2.5 × 10^4^/well) were cultured with irradiated splenocytes (3,000 rads, 2.0 × 10^5^ cells/well) in the presence of designated antigen concentrations. To measure GM-CSF activity, C57BL/6 bone marrow cells were cultured with designated concentrations of GM-CSF or GM-CSF fusion proteins. Cultures were pulsed with 1 μCi [^3^H]thymidine (6.7 Ci/mmol, New England Nuclear, Perkin Elmer, Waltham, MA, USA) during the last 24 h of a 72-h culture. Cultures were harvested onto filters by use of a Tomtec Mach III harvester (Hamden, CT, USA). [^3^H]thymidine incorporation into DNA was measured by use of a Perkin Elmer MicroBeta2 liquid scintillation counter. Error bars represented standard deviation of triplicate sets of wells.

### Induction and Assessment of EAE

CFA (Incomplete Freund's Adjuvant with 4 mg/ml heat-killed *Mycobacterium tuberculosis* H37Ra, BD Biosciences, Franklin Lakes, NJ) was mixed 1:1 with MOG35-55 in phosphate-buffered saline. The CFA/antigen mixture was emulsified by sonication. EAE was elicited by injection of 200 μg MOG35-55 in a total volume of 100 μl emulsion via three SC injections of 33 μl across the lower back. Each mouse received separate intraperitoneal injections (200 nanograms i.p.) of *Pertussis toxin* in PBS on days 0 and 2. All immunizations were performed under isoflurane anesthesia (Abbott Laboratories, Chicago, IL). Mice were assessed daily for clinical score and body weight. The following scale was used to score the clinical signs of EAE: 0, no disease; 0.5, partial paralysis of tail without ataxia; 1.0, flaccid paralysis of tail or ataxia but not both; 2.0, flaccid paralysis of tail with ataxia or impaired righting reflex; 3.0, partial hind limb paralysis marked by inability to walk upright but with ambulatory rhythm in both legs; 3.5, same as above but with full paralysis of one leg; 4.0, full hindlimb paralysis; 5.0, total hindlimb paralysis with forelimb involvement or moribund. A score of 5.0 was a humane endpoint for euthanasia.

EAE incidence was the number of EAE-afflicted mice compared to the total group size. Maximal scores were calculated as the most severe EAE score for each mouse. Mice that did not exhibit EAE had a score of zero, and these scores were included in the group average. Mice that exhibited humane endpoints as assessed by body weight loss, body score, or clinical score of 5.0 were subjected to humane euthanasia and were omitted from scoring thereafter. Time-course graphs portrayed daily mean maximal scores. Cumulative and maximal EAE scores were converted to ranked scores and analyzed by non-parametric ANOVA. To calculate percent maximal weight loss, 100% body weight was assigned as the maximal body weight obtained from day 1 through day 10, and daily body weights were calculated for each day after normalization to this 100% value. The minimum body weight was defined as the lowest body weight after normalization to the 100% value during the span of day 11 until the end of the experiment. Maximal weight loss was calculated by subtraction of the normalized minimum value from the 100% value. Negative weight loss values represented weight gain. Weight loss was analyzed by parametric ANOVA. Non-parametric and parametric ANOVA were assessed with a Bonferroni *Post Hoc* test unless noted otherwise. Incidence of EAE was analyzed pair-wise by Fisher's Exact Test. Mean EAE and weight loss data were shown with the standard error of the mean (SE).

### Preparation of GMCSF-MOG in Saline, Alum, and CFA

Vaccines containing GMCSF-MOG, GMCSF-OVA, GMCSF-NFM, GM-CSF, MOG35-55, or GM-CSF + MOG35-55 were administered at a dosage of either 2 or 4 nmoles as designated in the figure legends. CFA-based vaccines were prepared with equal parts of CFA and vaccine proteins/peptides (in PBS) for a total injection volume of 100 μl. The CFA/vaccine mixture was emulsified by sonication and injected via two SC injections of 50 μl across the back hindquarters. Conversely, vaccines in saline (no extrinsic adjuvant) were prepared in 200 μl of PBS and were administered SC by two injections of 100 μl each in the back hindquarters. Vaccines administered intravenously (IV) were given in 100 μl of PBS and injected retro-orbitally. Alum-based vaccines were prepared by mixing equal volumes of Alhydrogel adjuvant (InvivoGen) and vaccine proteins/peptides (in PBS) for a total injection volume of 150 μl per mouse. The Alum/vaccine mixture was incubated for 1 h on ice with continuous agitation to allow the protein/peptide to attach to the Alum gel. The vaccine was administered SC by two injections of 75 μl each in the back hindquarters.

## Results

### Depletion of FOXP3^+^ CD25^+^ Tregs With the Anti-CD25 mAb PC61 Reversed Tolerogenic Vaccination

**Figure 1:**
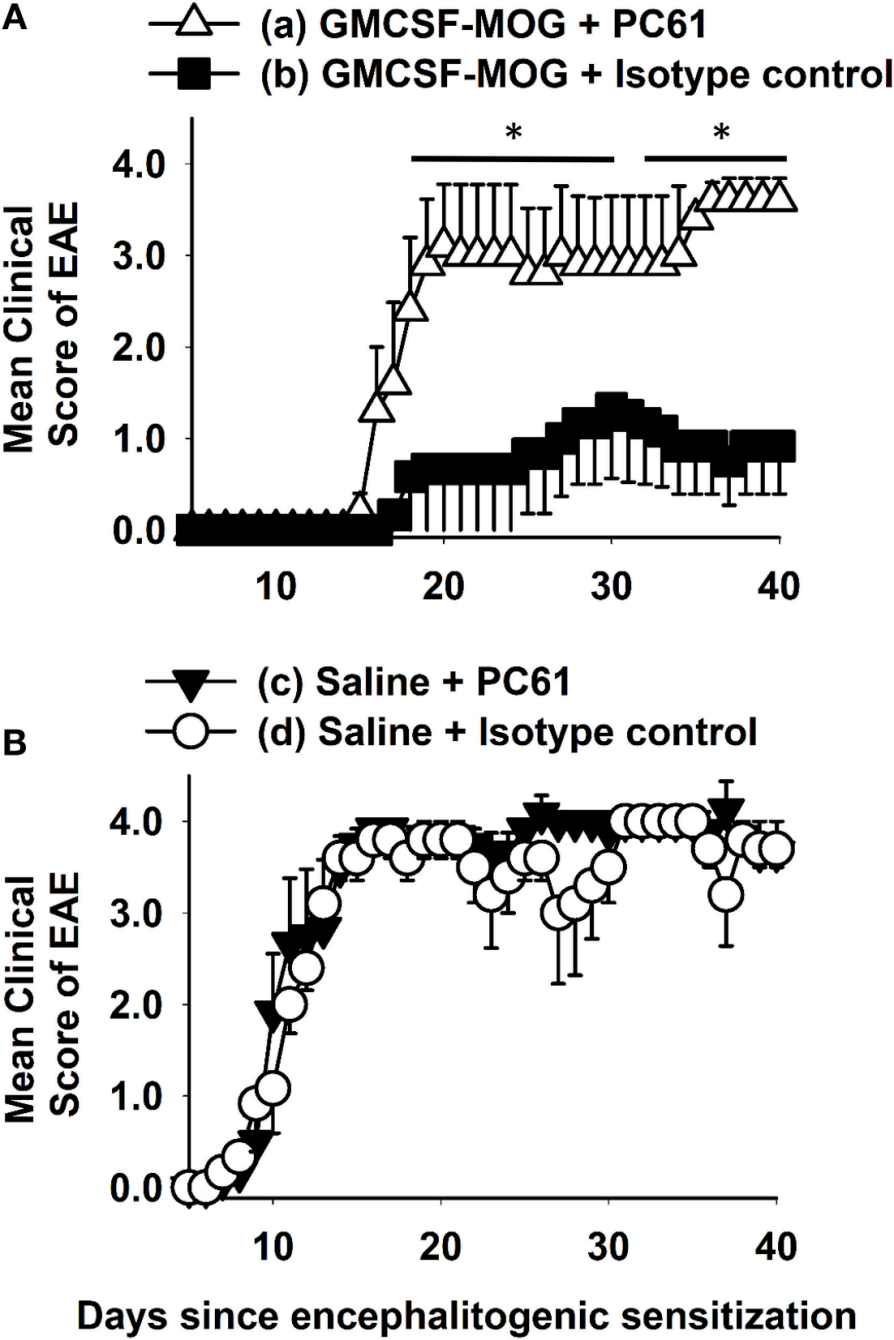
Depletion of CD25^+^ Tregs with the anti-CD25 mAb PC61 reversed tolerogenic vaccination. C57BL/6 mice were treated with 2 nmoles GMCSF-MOG **(A)** or saline **(B)** on days −21, −14, and −7. Mice were administered either 250 μg of the anti-CD25 PC61 mAb or 250 μg of a rat IgG1 isotype control mAb on days −4 and −2. On day 0, all mice were immunized with 200 μg MOG35-55 emulsified in CFA. Mice received 200 ng of Pertussis toxin i.p. on days 0 and 2. Shown are the daily mean clinical EAE scores through the end of the experiment on day 40 (*p < 0.05). Differences between (a) and (b) were analyzed using a two-way repeated measures ANOVA. Incidence of EAE was; (a) 5 of 5, (b) 3 of 6, (c) 6 of 6, and (d) 5 of 5. Mean maximal scores were; (a) 4.0 ± 0.0, (b) 1.3 ± 0.8, (c) 4.3 ± 0.2, and (d) 4.0 ± 0.0 (non-parametric ANOVA (b) vs. (a, c, d), *p* ≤ 0.012).

We hypothesized that GMCSF-based tolerogenic vaccines mediated tolerance via induction of CD25^high^ FOXP3^+^ Tregs (Figure 1). To address this question, we pretreated C57BL/6 with 2 nmoles GMCSF-MOG (A) or saline (B) on days −21, −14, and −7 and then administered the anti-CD25 PC61 mAb or a an IgG1 isotype control mAb on days −4 and −2 to deplete CD25^+^ FOXP3^+^ Tregs *in vivo* (59). Mice were then subjected to active induction of EAE on day 0. Pretreatment with the anti-CD25 PC61 mAb but not the isotype control antibody eliminated circulating CD25^+^ Tregs (data not shown). Pretreatment with the anti-CD25 PC61 mAb reversed the suppressive action of the tolerogenic vaccine such that the PC61-treated mice acquired full susceptibility to EAE and showed a chronic course of paralytic EAE equivalent to mice in the control groups (Figures 1A,B). PC61 had no effect on mice vaccinated with saline, presumably because Tregs played a minimal role in mice that were fully susceptible to EAE (Figure 1B). These results were mirrored by maximal weight loss: (a) 20.3% ± 4.6%, (b) 6.7% ± 4.2%, (c) 31.3% ± 1.0%, and (d) 26.4% ± 4.2%; b vs. c, d, *p* < 0.004. In conclusion, the GMCSF-MOG vaccine elicited CD25^+^ Tregs that were required for the inhibitory action of the tolerogenic vaccine. Thus, these data revealed a causal link between GMCSF-MOG vaccination, CD25^+^ Tregs, and tolerance induction in EAE.

### GMCSF-MOG Elicited a Robust FOXP3^+^ Treg Response in 2D2-FIG Mice

**Figure 2:**
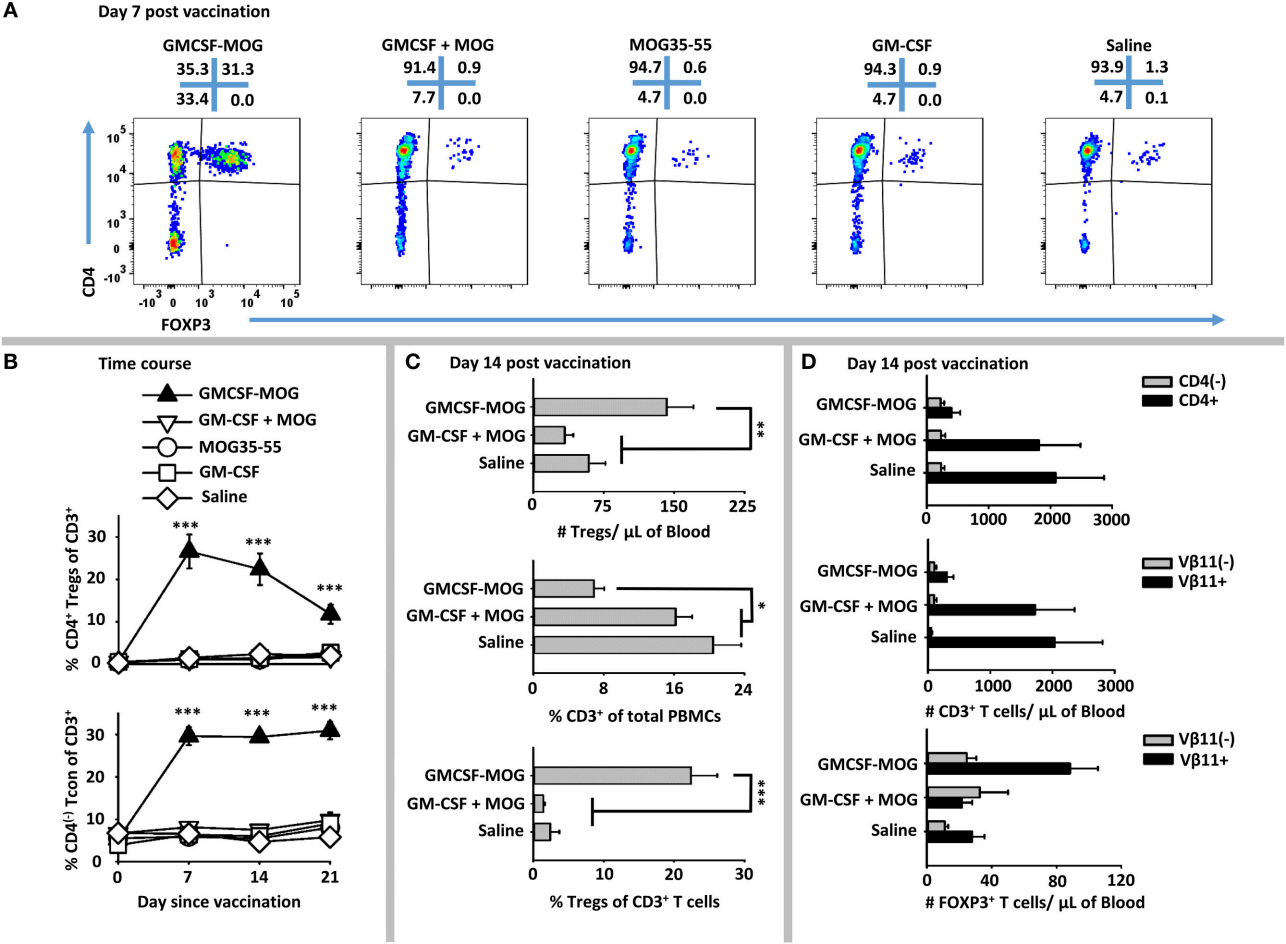
GMCSF-MOG elicited FOXP3^+^ Tregs in 2D2-FIG mice. On day 0, 2D2-FIG mice (*n* = 4/group) were vaccinated with GMCSF-MOG, the combination of GM-CSF + MOG35-55, MOG35-55 alone, GM-CSF alone, or saline. All injections were SC in saline at a dose of 4 nmoles. PBMCs were assayed for CD45, CD3, CD4, GFP (FOXP3), Vβ11 (2D2 TCRβ) by flow cytometry on days −1, 7, 14, and 21. Shown are **(A)** representative dot plots (day 7 time-point) from each treatment group gated on CD3^+^ T cells and analyzed for CD4 (y-axis) and FOXP3 (x-axis) expression. **(B)** The percentages of CD4^+^ FOXP3^+^ Tregs (top) and CD4^(−)^ FOXP3^(−)^ Tcon cells (bottom) among total CD3^+^ T cells are shown before vaccination (day 0) and for day 7, 14, and 21 time-points. Shown **(C)** are the total number of FOXP3^+^ Tregs per μl of blood (top) and percentages of CD3^+^ T cells among CD45^+^ leukocytes (middle) and FOXP3^+^ Tregs among CD3^+^ T cells (bottom) on day 14. Shown **(D)** are the number of CD3^+^ CD4^+^ and CD3^+^ CD4^(−)^ T cells per μl of blood (top) and the CD3^+^ Vβ11^+^ and CD3^+^ Vβ11^(−)^ T cells per μl of blood (middle) as well as the numbers of Vβ11^+^ FOXP3^+^ and Vβ11^(−)^ FOXP3^+^ Tregs (bottom) on day 14. Statistical significance was analyzed by use of a one-way ANOVA (*p < 0.05, **p < 0.01, ***p < 0.001). These data are representative of three independent experiments.

To address whether GMCSF-MOG expanded MOG-specific Tregs, we used 2D2-FIG mice that had a transgenic MOG-specific T cell repertoire and a GFP reporter of FOXP3 expression. 2D2-FIG mice were vaccinated SC with 4 nmoles of GMCSF-MOG, GM-CSF + MOG35-55, MOG35-55, GM-CSF in saline or with saline alone (Figure 2). A “day 0” baseline revealed that Tregs comprised <1.5% of all circulating T cells in naïve 2D2-FIG mice. By day 7 after vaccination with GMCSF-MOG, FOXP3^+^ Tregs comprised ~30% of all circulating T cells whereas mice vaccinated with control vaccines “GM-CSF + MOG35-55,” MOG35-55 alone, GM-CSF alone, or saline had baseline levels of Tregs (<1.5%) (Figure 2A). Previous research showed that covalent linkage of GM-CSF and NAg was required for the tolerogenic activity of GMCSF-MOG (30–32). Covalent linkage of GM-CSF to MOG35-55 was also required for induction of FOXP3^+^ Tregs (Figure 2A). Time course studies revealed that GMCSF-MOG vaccination increased Treg percentages to 27% of the circulating CD4^+^ T cell pool by day 7 and that significant percentages of these Tregs were maintained through days 14 (22%) and 21 (12%) (Figure 2B, top). Thus, after the initial vaccine-mediated inductive event, Treg percentages gradually attenuated throughout the remainder of the experiment.

GMCSF-MOG vaccination resulted in an increased percentage of CD4^(−)^ CD3^+^ Tcon cells, such that 30% of the circulating Tcon cells lacked CD4 expression as compared to 5% of T cells in control groups on days 7, 14, and 21 (Figure 2B, bottom). The absolute number of CD4^(−)^ T cells/μl of blood however was unchanged among all vaccine groups but the absolute number of CD4^+^ T cells was significantly diminished in mice that received GMCSF-MOG (~400/μl blood) as compared to control groups “GMCSF + MOG35-55” or saline (~2,000/μl of blood) (Figure 2D, top). These data indicate the GMCSF-MOG acted indirectly to increase percentages of CD4^(−)^ T cells by depleting CD4^+^ T cells rather than expanding the CD4^(−)^ T cell subset. Thus, GMCSF-MOG primarily affected the most reactive T cells (i.e., the CD4^+^ subset rather than the relatively non-reactive CD4^(−)^ subset).

GMCSF-MOG vaccination also reduced the percentages of circulating CD3^+^ T cells per total PBMCs by ~2.5 fold (~7% CD3^+^ T cells) compared to a baseline of 16-20% T cells in control groups that received “GM-CSF + MOG35-55” or saline (Figure 2C, middle). GMCSF-MOG selectively eliminated MOG- specific Vβ11^+^ (2D2 TCRβ) CD3^+^ Tcon cells (~300/μl of blood) as compared to control groups (~2,000/μl of blood), whereas Vβ11^(−)^ CD3^+^ T cells numbers remained unchanged (Figure 2D, middle). Thus, GMCSF-MOG exhibited antigen specificity by depleting NAg-reactive Vβ11^+^ T cells while sparing non-specific Vβ11^(−)^ T cells.

GMCSF-MOG vaccination resulted in ~140 Tregs/μl of blood compared to 50–60 Tregs/μl of blood in control groups (Figure 2C, top). GMCSF-MOG also selectively expanded the number of Vβ11^+^ Tregs compared to control groups whereas Vβ11^(−)^ Tregs numbers were unchanged (Figure 2D, bottom). These data indicated that at least two factors accounted for the elevated percentages of Tregs (Figure 2C, bottom), including an increase in the absolute numbers of circulating Tregs and a decrement in the absolute numbers of Tcon cells. Overall, these data indicate that GMCSF-MOG effectively targeted MOG35-55 to myeloid APC to expand MOG-specific CD4^+^ Tregs and deplete CD4^+^ MOG-specific Tcon cells while preserving CD4^(−)^ Tcon cells.

### GMCSF-MOG Elicited a System-Wide FOXP3^+^ Treg Lymphocytosis in Lymph Nodes, Spleen, and Blood

**Figure 3:**
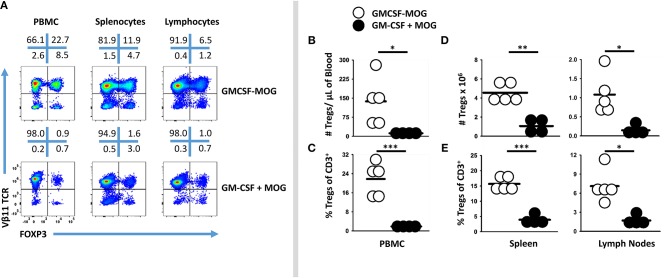
Subcutaneous administration of GMCSF-MOG in saline elicited FOXP3^+^ Tregs in lymph nodes, spleen, and blood. On day 0, 2D2-FIG mice (*n* = 4–5/group) were vaccinated with 4 nmoles of GMCSF-MOG or 4 nmoles GM-CSF + 4 nmoles MOG35-55. PBMC were analyzed on day 4, and draining inguinal lymph nodes and spleen were analyzed on days 5 and 6. **(A)** Representative dotplots of CD3 gated T cells from the peripheral blood, lymph nodes, and spleen were analyzed for Vβ11 (2D2 TCRβ) (y-axis) and FOXP3 (x-axis). Shown are numbers of Tregs per μl of blood **(B)**, percentages of Tregs among the total CD3^+^ T cells **(C)**, as well as the total numbers **(D)** and percentages **(E)** of Tregs among the total CD3^+^ T cells in the spleen and lymph nodes. Each dot represents a single mouse. The bar represents the mean. Statistical significance was analyzed by use of a one-tailed *t*-test (*p < 0.05, **p < 0.01, ***p < 0.001). These data are representative of three independent experiments.

GMCSF-MOG primed a system-wide Treg response in that the vaccine elicited high frequencies of FOXP3^+^ Tregs in the spleen, draining inguinal lymph nodes, and blood (Figure 3A). 2D2-FIG mice were vaccinated with GMCSF-MOG or “GMCSF + MOG35-55” on day 0, PBMC were analyzed on day 4, and lymphoid organs were analyzed on days 5 and 6. GMCSF-MOG vaccination induced high percentages of FOXP3^+^ Tregs in all three compartments including ~22, 15, and 7% of all T cells in PBMC, spleen, and lymph nodes, respectively (Figures 3A,C,E). Mice that received the control vaccine “GM-CSF + MOG35-55” had relatively low frequencies of FOXP3^+^ Tregs (~1, 4, and 2% Tregs in PBMC, spleen, and lymph nodes, respectively). GMCSF-MOG increased the total number of Tregs as compared to the “GM-CSF + MOG35-55” control vaccine (Figures 3B,D). GMCSF-MOG vaccination resulted in ~4.5 × 10^6^ Tregs as compared to 1.0 × 10^6^ Tregs per spleen in “GM-CSF + MOG” vaccinated mice. Similarly, GMCSF-MOG induced ~1.2 × 10^6^ Tregs in the inguinal lymph nodes as compared to 0.2 × 10^6^ Tregs in control nodes. These results indicate that SC vaccination with GMCSF-MOG in saline elicited Treg responses throughout the secondary lymphoid organs and the circulation.

### Booster Vaccination of GMCSF-MOG Maintained Circulating Levels of FOXP3^+^ Tregs

**Figure 4:**
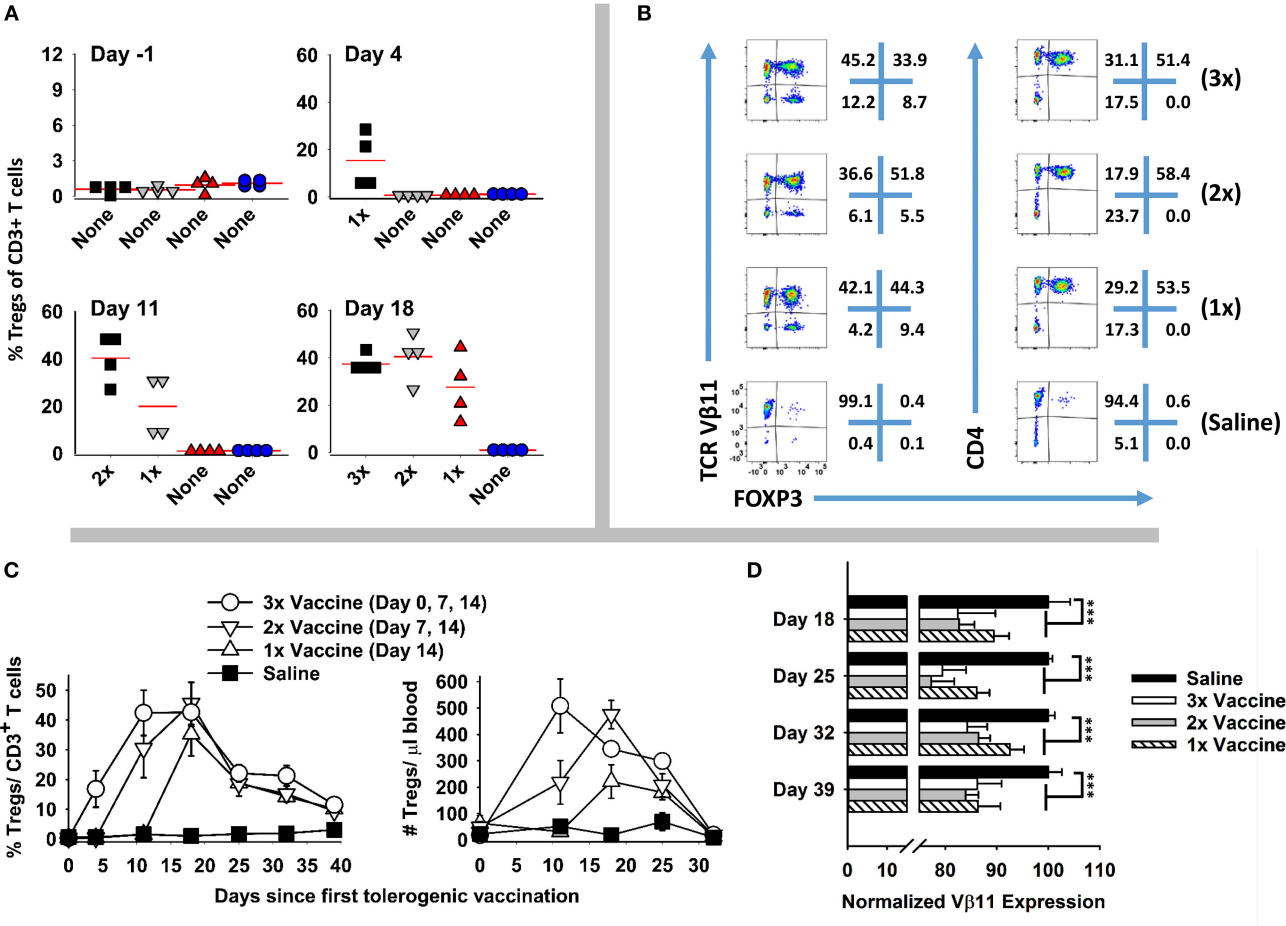
Booster vaccines with GMCSF-MOG maintained circulating levels of Tregs. 2D2-FIG mice were injected with 4 nmoles of GMCSF-MOG or saline on days 0, 7, and 14. One group received three GMCSF-MOG vaccinations (3x), one group received saline on day 0 and vaccine on days 7 and 14 (2x), one group received saline on days 0 and 7 and vaccine on day 14 (1x), and one group received saline on days 0, 7, and 14 (*n* = 4/group). T cells in peripheral blood were assayed for CD45, CD3, CD4, GFP (FOXP3), Vβ11 (2D2 TCRβ) expression on days −1, 4, 11, 18, 25, 32, and 39. **(A)** PBMC were assessed for percentages of FOXP3^+^ Tregs among total CD3^+^ T cells on day −1, 4, 11, and 18. **(B)** For day 18, Vβ11 (y-axis) or CD4 (y-axis) expression is shown as a function of FOXP3 expression (x-axis) among CD3^+^ T cells. **(C)** Percentages and numbers (per μl blood) of FOXP3^+^ Tregs are shown for CD3^+^ T cells collected on days −1, 4, 11, 18, 25, 32, and 39. For % Tregs, the 1x, 2x, and 3x groups were significantly different from saline on days 18, 25, 32, and 39. For numbers of Tregs/μl of blood, significant differences were noted on days 18 and 25 (*p* < 0.05). **(D)** The MFI of Vβ11 expression among Vβ11^+^ T cells is shown for PBMC samples collected on days 18, 25, 32, and 39. Statistical significance was analyzed by use of a one-way ANOVA (***p < 0.001).

Booster immunizations were used to assess whether repeated immunization of GMCSF-MOG elicited sustained Treg responses. 2D2-FIG mice were given three injections (days 0, 7, 14), two injections (days 7, 14), or one injection (day 14) of GMCSF-MOG (Figure 4). On day −1, baseline FOXP3^+^ Tregs as a percentage of total CD3^+^ T cells in PBMC were <1.5% for all 16 mice (Figure 4A). Vaccination with GMCSF-MOG elicited circulating FOXP3^+^ Tregs by day 4 with a range of 5–29%. By day 11, mice receiving 2 immunizations exhibited percentages of Tregs ranging from 27 to 49% of all circulating T cells whereas mice receiving 1 immunization exhibited Treg percentages ranging from 6 to 32%. Percentages of circulating Tregs on day 18 ranged from 26 to 42% (3 injections, 3x), 33-50% (2 injections, 2x), 12–44% (single injection, 1x), and ~1% for saline treated mice. Major FOXP3^+^ subpopulations were noted in both transgenic Vβ11^+^ T cells and non-transgenic Vβ11^(−)^ populations whereas FOXP3^+^ T cells were exclusively CD4^+^ (Figure 4B). Major populations of Vβ11^(−)^ Treg were attributed to the overall loss of CD3^+^ Tcon cells, resulting in elevated percentages but not numbers of Vβ11^(−)^ Tregs. Elevated percentages of FOXP3^+^ T cells in vaccinated mice mirrored increased numbers of FOXP3^+^ T cells per μl of blood (Figure 4C). As measured by FOXP3^+^ Treg frequencies or absolute numbers, the disappearance of circulating Tregs occurred at similar rates in the 3×, 2×, and 1× vaccine groups. Thus, multiple boosters maintained Tregs in circulation during repeated immunizations. For example, the 3x vaccination group showed a longer duration of Treg presence in the circulation compared to the 1x vaccination group. However, after the last vaccination on day 14, Tregs disappeared from the blood at similar rates during the next 2–3 weeks.

GMCSF-MOG (1x, 2x, and 3x) resulted in the down-regulation of TCRα/β on a per cell basis (Figure 4D). Diminished expression of TCR Vβ11 expression however did not rebound to baseline levels during this time span. Rather, lower levels of TCR-Vβ11 expression instead appeared to represent a new set-point for the 2D2 repertoire. These findings indicated that GMCSF-MOG not only elicited a major FOXP3^+^ Treg population but also desensitized T cell antigen recognition among the Tcon repertoire.

### GMCSF-MOG Induced a FOXP3^+^ Population With a Canonical Treg Phenotype

**Figure 5:**
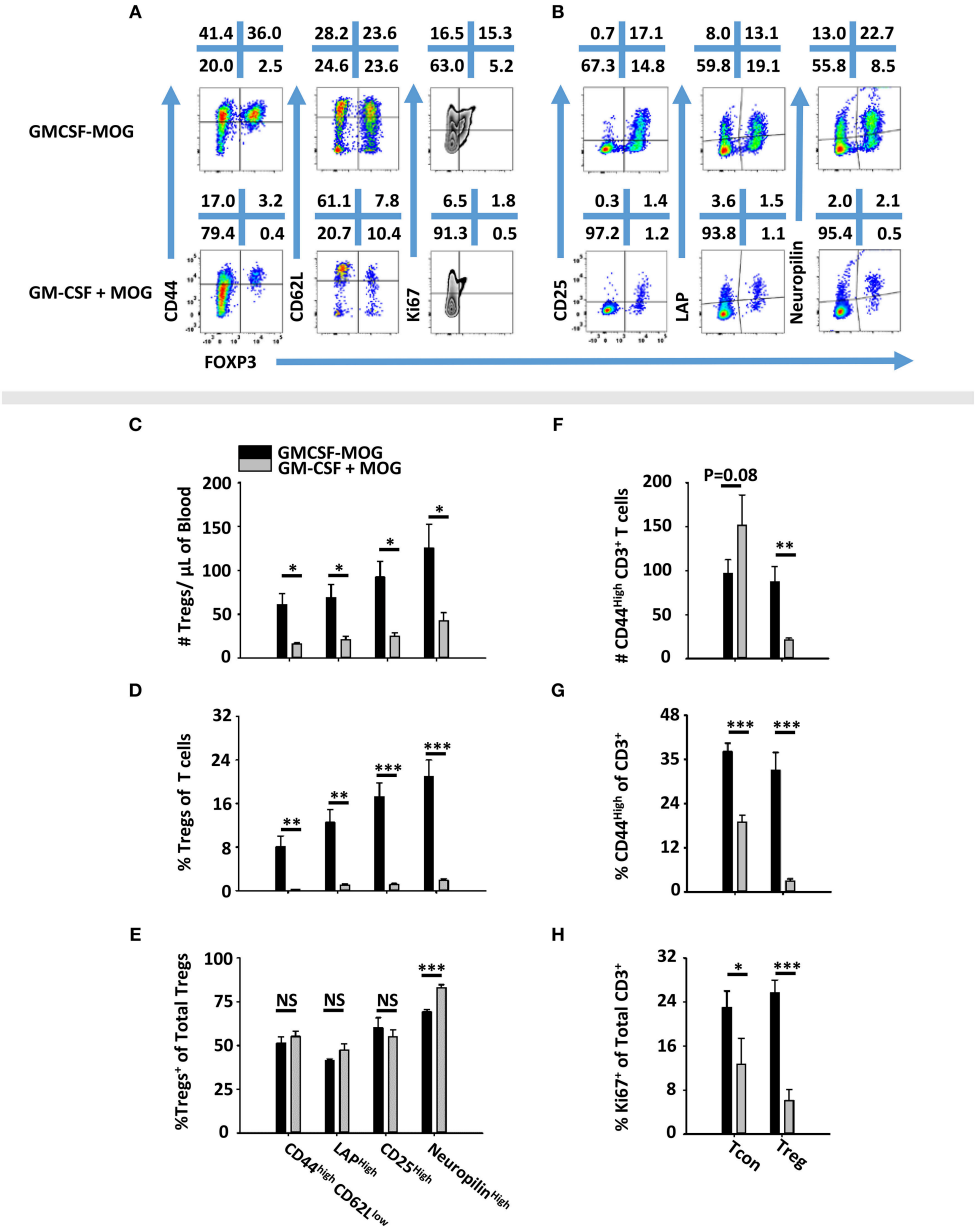
GMCSF-MOG induced a FOXP3^+^ T cell population with a canonical Treg phenotype. On day 0, 2D2-FIG (*n* = 4–5/group) mice were vaccinated subcutaneously with 4 nmoles of GMCSF-MOG or 4 nmoles GM-CSF + 4 nmoles MOG35-55. PBMCs were analyzed on day 4. Shown **(A)** are representational dotplots of CD3^+^ T cells analyzed for CD44, CD62L, and Ki67 expression (y-axis) and **(B)** CD4^+^ T cells analyzed for CD25, LAP, and Neuropilin vs. FOXP3 expression (x-axis). Shown are numbers of Tregs per μl of blood **(C)** or percentages of Tregs **(D)** that express CD44^high^ CD62L^low^, LAP, CD25, or Neuropilin among total T cells **(D)** or among Tregs **(E)**. Shown are the Treg and Tcon cell numbers per μl of blood **(F)** and percentages of CD44^high^ Tcons or Tregs among CD3^+^ T cells **(G)**. Shown are **(H)** percentages of Ki67^+^ Tregs or Tcons among CD3^+^ T cells. Statistical significance was analyzed by use of a one-tailed *t*-test (*p < 0.05, **p < 0.01, ***p < 0.001). These data are representative of two independent experiments.

To determine the phenotype of GMCSF-MOG-induced Tregs, 2D2-FIG mice were vaccinated SC with GMCSF-MOG or “GM-CSF + MOG35-55” in saline. PBMC were analyzed on day 4 for CD44, CD62L, CD25, LAP, Neuropilin, and Ki67 expression (Figures 5A,B). GMCSF-MOG vaccinated mice exhibited significantly increased numbers and percentages of CD44^high^ CD62L^low^, LAP^high^, CD25^high^, and Neuropilin^high^ Tregs in blood compared to control mice (Figures 5C,D). For example, in GMCSF-MOG vaccinated mice, ~8% of total T cells expressed a FOXP3^+^ CD44^high^-CD26L^low^ phenotype compared to ~1% in control mice (Figure 5D). GMCSF-MOG vaccination also increased the percentages of T cells that expressed a FOXP3^+^, LAP^high^ (14%), CD25^high^ (18%), or Neuropilin^high^ (22%) phenotype compared to control mice in which <2% of the T cells were Tregs positive for any of the respective markers (Figure 5D). Interestingly, the two treatment groups did not differ in the percentages of Tregs that expressed these markers within the Treg pool, aside from a modest decrease (~13 %) in Neuropilin^high^ Tregs in GMCSF-MOG treated mice (Figure 5E). GMCSF-MOG vaccination also increased the number and percentages of T cells that were CD44^high^ Tregs compared to control mice (Figures 5F–G), whereas GMCSF-MOG vaccination increased percentages but not numbers of T cells that were CD44^high^ Tcon cells, a discrepancy that reflected the generalized loss of Tcon cells. GMCSF-MOG vaccination increased both Treg and Tcon intracellular staining for Ki67, a marker of cell division, compared to control mice although Ki67 expression was upregulated 5-fold in Tregs vs. 2-fold in Tcon cells (Figure 5H). These data showed that GMCSF-MOG-induced Tregs have a phenotype similar to preexisting Tregs and that GMCSF-MOG favors the expansion of MOG-specific Tregs over MOG-specific Tcon cells.

### The Antigenic Domain of GMCSF-Antigen Fusion Proteins Was a Major Parameter for Treg Induction

**Figure 6:**
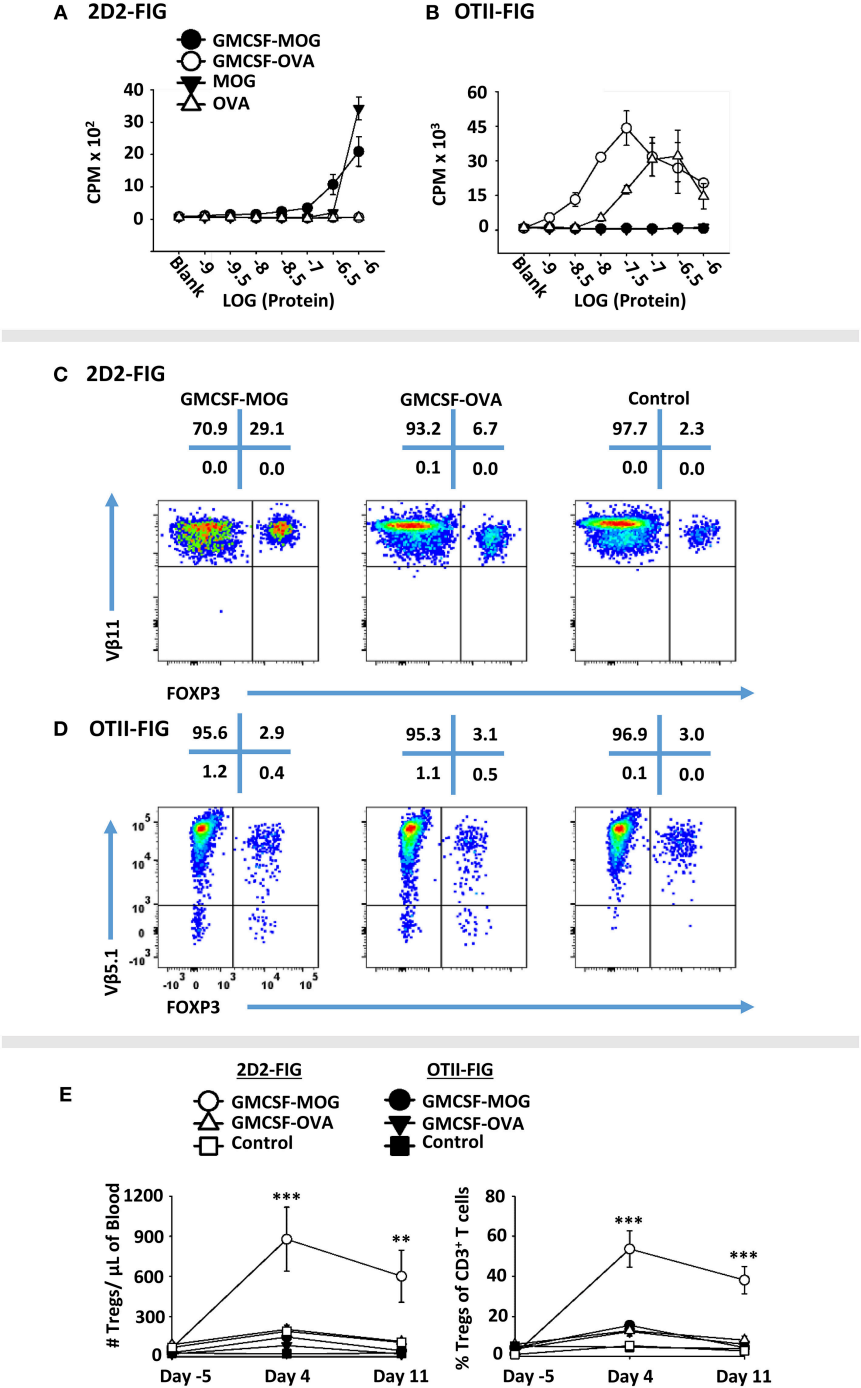
GMCSF-MOG Treg induction was dependent upon the antigenic domain. Twenty-five thousand 2D2 T cells **(A)** or OTII T cells **(B)** from continuous T cell lines were cultured with 100,000 irradiated splenocytes and designated concentrations (x-axis) of GMCSF-MOG, GMCSF-OVA, MOG35-55, or OVA323-339. Cultures were pulsed with 1 μCi of [^3^H]thymidine during the last 24 h of a 3-day culture, and counts per minute (CPM, y-axis) were measured on day 3. **(C–E)** On day 0, 2D2-FIG or OTII-FIG (*n* = 3–4/group) were subcutaneously vaccinated with either 4 nmoles of GMCSF-MOG, 4 nmoles GMCSF-OVA, or with saline (OTII-FIG) or GM-CSF + MOG35-55 (2D2-FIG). PBMCs were assayed on days −5, 4, and 11 for CD3, CD4, Vβ11 (2D2 TCRβ), Vβ5.1 (OTII TCRβ) and FOXP3 expression. Representative dotplots of 2D2-FIG **(C)** and OTII-FIG **(D)** PBMCs were analyzed for Vβ11 or Vβ5.1 (y-axis) respectively and FOXP3 (x-axis) among CD3^+^ T cells. Shown **(E)** are Treg numbers per μl of blood and Treg percentages of total CD3^+^ T cells on days −5, 4, and 11. Statistical significance was analyzed by use of a one-way ANOVA (**p < 0.01, ***p < 0.001). These data are representative of three independent experiments.

A major question was whether the GM-CSF domain or the antigenic domain of GMCSF-antigen fusion proteins represented the predominant variable polarizing T cells into the Treg lineage. To assess this issue, GMCSF-MOG, and GMCSF-OVA were compared for Treg induction in MOG-specific (2D2-FIG) vs. OVA-specific (OTII-FIG) mice. GMCSF-MOG and GMCSF-OVA were exquisitely specific in stimulating proliferation by MOG-specific T cells and OVA-specific T cells, respectively (Figures 6A,B). As expected, given that MOG represented a self-antigen and OVA represented a foreign antigen in mice, the antigenic activity of GMCSF-MOG and MOG35-55 in 2D2-FIG T cell cultures was substantially less potent than the respective GMCSF-OVA and OVA323-339 responses of OTII-FIG T cells. That is, 2D2 T cell responses to GMCSF-MOG and MOG35-55 were at least 100-fold less potent than those of OTII T cells to GMCSF-OVA and OVA323-339, respectively. Thus, MOG35-55 represented a low-affinity or inefficient T cell epitope whereas OVA323-339 represented a relatively high-affinity, high-efficiency T cell epitope in the respective systems. Both GMCSF-MOG and GMCSF-OVA displayed enhanced antigen potency as compared to their respective peptide counterparts, MOG35-55 and OVA323-339, which most likely reflected antigenic targeting to myeloid APC via GM-CSF and the GM-CSF receptor (CD116, CD131).

To assess induction of Tregs, GMCSF-MOG, GMCSF-OVA, or control vaccines GM-CSF (2D2-FIG) or saline (OTII-FIG) were used to vaccinate 2D2-FIG or OTII-FIG mice (Figures 6C–E). As expected, GMCSF-MOG vaccination caused a significant induction of absolute numbers and percentages of MOG-specific Tregs in 2D2-FIG mice (Figure 6C), including an average of 55% Tregs per the total CD3^+^ T pool by day 4 (Figure 6E, right panel). GMCSF-MOG however did not significantly elicit Tregs in OTII-FIG mice, which verified that specific T cell antigen recognition was a requirement for induction of Tregs (Figure 6D). In contrast, GMCSF-OVA vaccination of OTII-FIG or 2D2-FIG mice did not reliably elicit significant increases in the absolute numbers or percentage of Tregs compared to control mice (Figures 6C,D). These data indicated that specific T cell antigen recognition, although required in the GMCSF-MOG/2D2-FIG system, was not sufficient for induction of Tregs in the GMCSF-OVA/OTII-FIG system. These findings indicated that T cell antigen recognition was necessary but not sufficient for induction of Tregs. Rather, the quality of T cell antigen recognition was the critical parameter polarizing the Treg/Tcon balance, in that the low efficiency ligand MOG35-55 in GMCSF-MOG was best adapted to support induction of Tregs. These experiments revealed that intrinsic qualities of the antigen covalently attached to the GM-CSF fusion partner played a key role in Treg induction.

The GM-CSF domain of GMCSF-MOG was also critical for induction of Tregs because the synthetic MOG35-55 peptide did not independently elicit Tregs (Figures 2, 3, 5). Rather, the covalently-linked GM-CSF and MOG35-55 domains were both required for efficient induction of Tregs. Interestingly, GM-CSF alone (i.e., GM-CSF, GMCSF-MOG in OTII mice, GMCSF-OVA in 2D2 mice) resulted in increased numbers and percentages of Tregs (2–10%) as compared to saline (<2% Tregs). However, these increases were transient (day 4) and were modest when compared to the effect of GMCSF-MOG vaccine in 2D2-FIG mice (Figure 6E). These findings are consistent with previous studies showing that GM-CSF alone increased Treg proliferation in rodent models of autoimmune disease (48, 50, 51, 53, 54).

### Induction of Tregs by GMCSF-MOG Was Associated With Inefficient TCR Ligation

**Figure 7:**
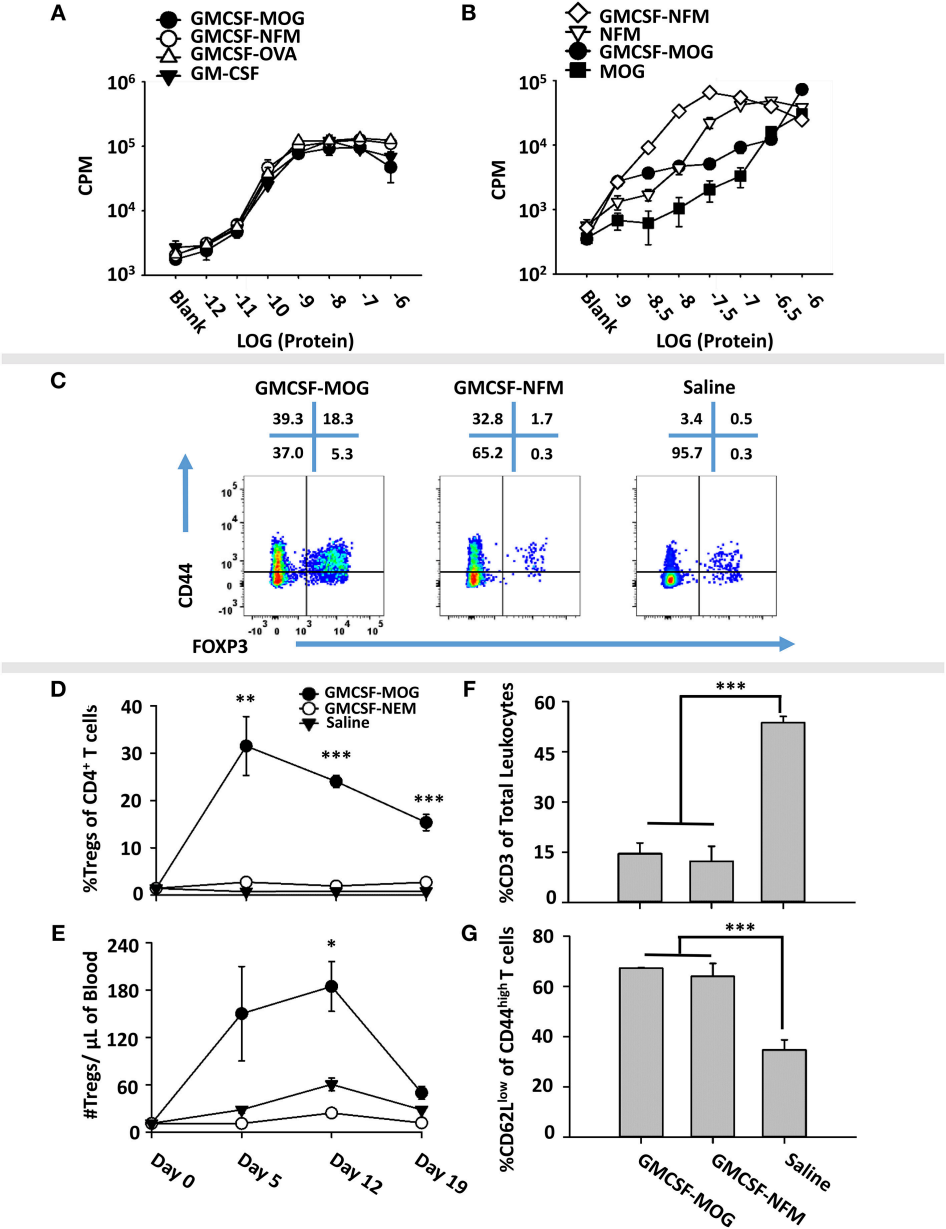
Induction of Tregs by GMCSF-MOG was associated with inefficient TCR ligation. **(A)** Designated concentrations (x-axis) of GM-CSF, GMCSF-MOG, GMCSF-NFM, or GMCSF-OVA were incubated with 100,000 C57BL/6 bone marrow cells for 3 days. **(B)** 25,000 2D2 T cells were cultured with 100,000 irradiated splenocytes with designated concentrations (x-axis) of GMCSF-MOG, GMCSF-NFM, MOG35-55, or NFM13-37. **(A,B)** Cultures were pulsed with 1 μCi of [^3^H]thymidine during the last 24 h of a 3-day culture. **(C–G)** On day 0, 2D2-FIG mice were subcutaneously vaccinated with either 4 nmoles of GMCSF-MOG (*n* = 5), 4 nmoles GMCSF-NFM (*n* = 3), or saline (*n* = 3). PBMCs were assayed on 5, 12, and 19 for CD3, CD4, Vβ11 (2D2 TCRβ), CD44, CD62L, and GFP (FOXP3) expression. The pre-day 0 bleed was derived from the average Treg percentages among CD4^+^ T cells and the average number of Tregs per μl of blood (*N* = 50 mice from 3 independent experiments). **(C)** On day 12, PBMCs from GMCSF-MOG, GMCSF-NFM, or saline treated mice were analyzed for CD44 (y-axis) and FOXP3 (x-axis) among CD3^+^ CD4^+^ T cells. Shown **(D,E)** are Treg numbers per μl of blood and Treg percentages among CD4^+^ T cells on days 5, 12, and 19. Shown are percentages of CD3^+^ T cells among total CD45^+^ leukocytes **(F)** and percentages of CD62L^low^ cells among CD44^high^ CD3^+^ CD4^+^ T cells **(G)** on day 12. Statistical significance was analyzed by use of a one-way ANOVA (*p < 0.05, **p < 0.01, ***p < 0.001). These data are representative of three independent experiments.

To test whether low-efficiency TCR ligands are optimal for induction of Tregs, we devised an alternative experimental system based on the observation that the 2D2 TCR recognizes two distinct NAg, including MOG35-55 as a low affinity antigen and NFM13-37 as a high affinity antigen (60, 61). We derived an expression system for GMCSF-NFM, which exhibited GM-CSF activity equivalent to that of GMCSF-MOG, GMCSF-OVA and GM-CSF in bone marrow proliferation assays (Figure 7A). Each recombinant protein induced equivalent proliferation responses with a half-maximal stimulation in the 10–100 picomolar range. These assays confirmed that C-terminal antigenic domains did not affect potency of the GM-CSF cytokine. To measure activity of the antigenic domain, GMCSF-MOG, GMCSF-NFM, MOG35-55, and NFM13-37 were compared in 2D2 T cell proliferative assays (Figure 7B). GMCSF-NFM exhibited the highest potency (half-maximal stimulation at ~3.2 − 10 nM). GMCSF-NFM was ~10-fold more potent that NFM13-37 and was several orders of magnitude more active than either GMCSF-MOG or MOG35-55.

To assess induction of Tregs, GMCSF-MOG, GMCSF-NFM, or saline were used to vaccinate 2D2-FIG mice, and PBMCs were analyzed on day 4 for Treg induction. GMCSF-MOG induced high percentages of FOXP3^+^ Tregs in the CD4^+^ T cell pool. Importantly, GMCSF-NFM lacked activities necessary for induction of Tregs (~1%) (Figure 7C). GMCSF-MOG induced a sustained Treg response as shown by high percentages of Tregs on day 5 (30%), day 12 (25%), and day 19 (20%) and increased numbers of Tregs/μl of blood on day 5 (130/μl) and day 12 (180/μl). In contrast, GMCSF-NFM did not affect Treg numbers or percentages during the 19 days of the experiment (Figures 7D,E). These data support the hypothesis that GMCSF-antigen fusion proteins containing high-efficiency TCR ligands lack activities required for the robust induction of FOXP3^+^ Tregs. Although GMCSF-MOG and GMCSF-NFM differed qualitatively in activities needed for the induction of Tregs, both vaccines caused activation of the 2D2-FIG T cell repertoire as shown by the increased percentages of CD62L^low^ CD44^high^ T cells (Figure 7G). Both vaccines also caused the diminution of the 2D2 Tcon repertoire (Figure 7F), which may represent a separate mechanism of tolerance.

### The Treg-Inductive Activity of GMCSF-MOG Remained Intact When Administered in Pro-immunogenic Adjuvants

Previous studies showed that GMCSF-MOG and GMCSF-PLP(139–151) imposed tolerogenic outcomes at relatively low doses even when emulsified with the respective encephalitogenic peptide in CFA in the C57BL/6 and SJL models of EAE (29). These data indicated that GMCSF-NAg exerted operational tolerance even in strong pro-inflammatory environments. For example, inclusion of 1 nmole GMCSF-MOG with 77.5 nmoles of MOG35-55 in CFA strongly inhibited incidence, severity, and duration of EAE compared to mice immunized with the CFA/MOG35-55 emulsion without GMCSF-MOG. These data raised the important question of whether GMCSF-MOG would retain Treg inductive capacity in adjuvant primed proinflammatory environments.

**Figure 8:**
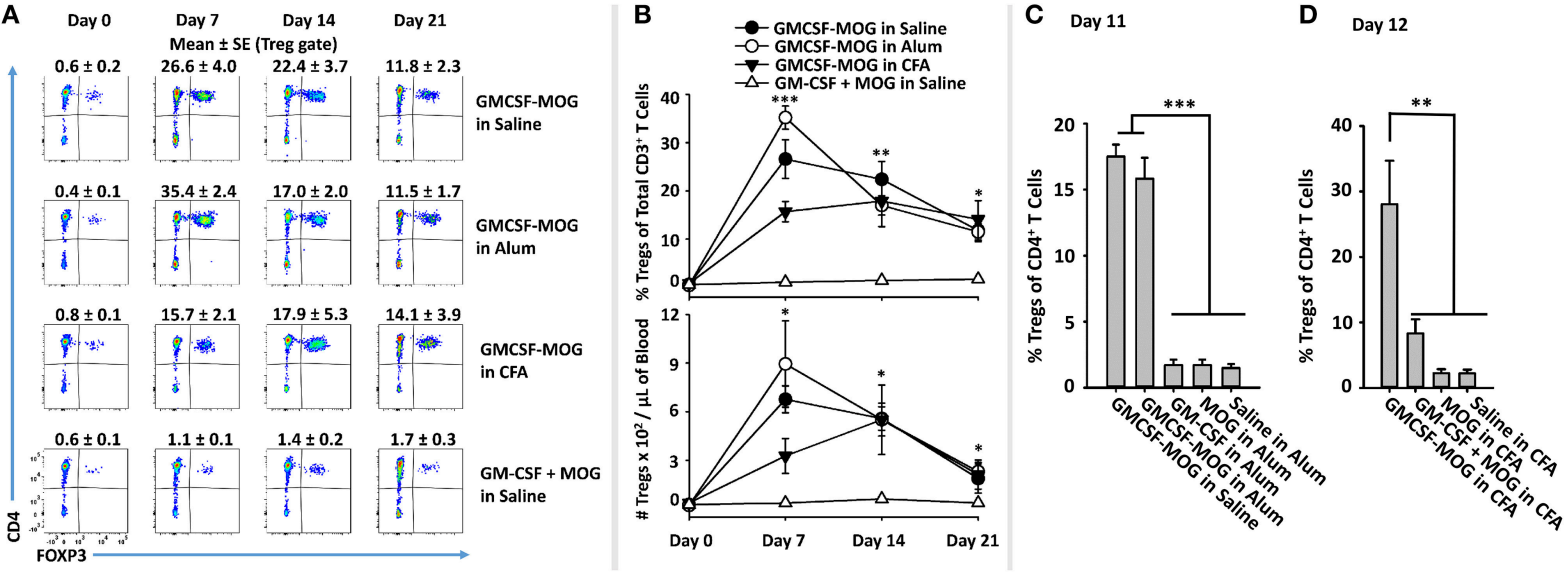
The Treg-inductive activity of GMCSF-MOG remained intact when administered in pro-immunogenic adjuvants. **(A,B)** On day 0, 2D2-FIG mice (*n* = 4/group) were or were not vaccinated with GMCSF-MOG in saline, Alum, or CFA, and PBMCs were obtained on days 0, 7, 14, and 21. All injections were SC at a dose of 4 nmoles. **(A)** CD3^+^ T cells were analyzed for CD4 (y-axis) and FOXP3 (x-axis) expression. Percentages of CD4^+^ FOXP3^+^ Tregs are designated at the top of each dotplot. Shown **(B)** are percentages of FOXP3^+^ Tregs among CD3^+^ T cells (top) and Treg numbers per μl of blood (bottom) on days 0, 7, 14, and 21. **(C)** On day 0, 2D2-FIG mice (*n* = 4/group) were vaccinated with 4 nmoles of GMCSF-MOG, GM-CSF, MOG35-55, or saline in 100 μl of Alum, and blood was analyzed on day 11 for Treg percentages among CD4^+^ T cells. **(D)** On day 0, 2D2-FIG mice (*n* = 4/group) were vaccinated with 4 nmoles of GMCSF-MOG, GM-CSF + MOG, MOG35-55, or saline in CFA and blood was analyzed on day 12 for Treg percentages among CD4^+^ T cells. Statistical significance was analyzed by use of a one-way ANOVA (*p < 0.05, **p < 0.01, ***p < 0.001). These data are representative of three independent experiments.

To assess this question, GMCSF-MOG was prepared in saline, Alum, or CFA and injected into 2D2-FIG mice (Figures 8A,B). Alum vaccine formulations incorporated GMCSF-MOG, GM-CSF, MOG35-55, or saline into the Alum adjuvant. A single vaccination of GMCSF-MOG/CFA, GMCSF-MOG/Alum, and GMCSF-MOG in saline elicited high percentages of circulating Tregs that persisted through the 21-day assessment (Figure 8B). On day 7, the GMCSF-MOG/Alum and GMCSF-MOG/saline vaccines elicited higher Treg percentages than GMCSF-MOG/CFA although these differences disappeared by days 14 and 21 (Figure 8B). On day 11, GMCSF-MOG in saline and GMCSF-MOG/Alum both elicited high percentages of Tregs while GMCSF/Alum, MOG35-55/Alum, and saline/Alum did not elicit significant Treg responses (Figure 8C). These findings are consistent with the concept that low-efficiency T cell antigen recognition events are integrated within the confines of an immunological synapse and can support Treg induction without regard to local inflammatory environmental cues.

A related question was whether incorporation of GMCSF-MOG into CFA would relieve the strict requirement for covalent coupling of GM-CSF and NAg, because the two domains would be physically sequestered in the same antigenic depot. Vaccine formulations in CFA included GMCSF-MOG, “GMCSF + MOG35-55,” MOG35-55, or saline in CFA. GMCSF-MOG/CFA induced a significant Treg response whereas the other CFA-based vaccine formulations including “GMCSF + MOG35-55” in CFA lacked robust Treg inductive capability (Figure 8D). These data indicate that GMCSF-MOG exerted a dominant Treg response even within the context of a CFA adjuvant-primed lymphatic drainage. However, co-localization of independent GM-CSF and NAg molecules in the same adjuvant-based antigenic depot did not relieve the requirement for covalent cytokine-NAg linkage. These data suggest that a continued requirement of GMCSF-NAg linkage is needed for Treg induction during and/or after the immunological processing of the CFA antigenic depot.

### Subcutaneous and Intravenous Routes of GMCSF-MOG Administration Drove Robust Treg Responses

**Figure 9:**
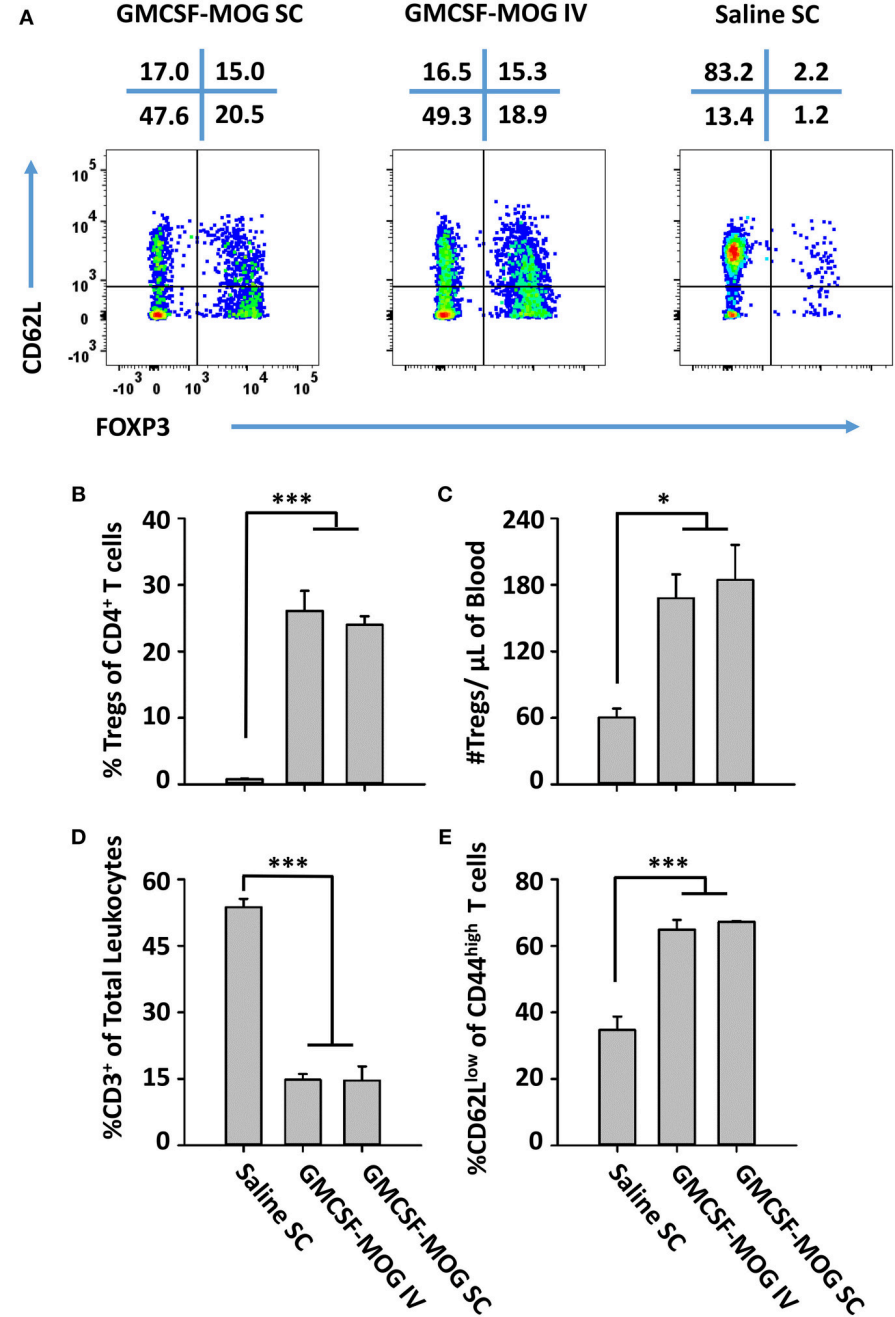
GMCSF-MOG induced Tregs when administered intravenously. On day 0, 2D2-FIG mice were vaccinated with 4 nmoles of GMCSF-MOG intravenously via the retro-orbital route (*n* = 5) or by SC (*n* = 5) injection or were vaccinated SC with saline alone (*n* = 3). Blood was analyzed on day 12. **(A)** CD3^+^ CD4^+^ CD44^high^ T cells were analyzed for CD62L expression (y-axis) and FOXP3 (x-axis). The percentages of each quadrant are designated at the top of each dotplot. Shown **(B)** are the percentages of FOXP3^+^ Tregs among total CD3^+^ CD4^+^ T cells and **(C)** total numbers of FOXP3^+^ Tregs per μl of blood. Shown **(D)** are percentages of CD3^+^ T cells among total leukocytes in the blood. Shown **(E)** are the percentages of CD62L^low^ T cells among CD44^high^ CD3^+^ CD4^+^ T cells. Mean percentages of CD44^+^ T cells in the CD3^+^ CD4^+^ T cell pool for the “GMCSF-MOG IV” and “GMCSF-MOG SC” groups (61% ± 4%, 54% ± 4%, respectively) were significantly different from those for the saline group (6% ± 1%) (*p* ≤ 0.001). Statistical significance was analyzed by use of a One-way ANOVA (*p < 0.05, ***p < 0.001). These data are representative of two independent experiments.

The observation that SC GMCSF-MOG vaccination elicited the highest Treg frequencies in the blood (30–40%) rather than the spleen (13–15%) or lymph nodes (6%) raised questions whether the Treg inductive response required a classical lymphatic drainage (Figure 3). The expectation was that SC injection of GMCSF-MOG would target MOG35-55 to myeloid APC in the draining lymphatics at the site of inoculation and that sensitization of the Treg response would occur in the draining lymphatics. Conversely, IV administration of GMCSF-MOG would predictably bypass the lymphatic drainage and instead introduce NAg directly into the blood, spleen, lung, and liver. To assess this question, the optimal route of GMCSF-MOG administration was tested by comparing SC or IV injections in 2D2-FIG mice. Notably, either route was equally effective. Administration of GMCSF-MOG via either SC and IV route caused robust Treg responses marked by ~25% Tregs (of the total CD4^+^ T cell population) as compared to mock (saline) vaccination (3% Tregs) when assessed on day 12 (Figures 9A,B). Both SC and IV vaccination also resulted in increased percentages of activated 2D2 T cells marked by high expression levels of CD44 and low levels of CD62L (Figures 9A,E). Although both SC and IV administration of GMCSF-MOG caused the activation of both Treg and Tcon subsets, the vaccine preferentially drove the expansion Tregs in both cases. Mice vaccinated by either route had significantly increased levels of Treg per μl of blood (~180) as compared to saline (~60) (Figure 9C) and reduced percentages and numbers of circulating CD3^+^ T cells (~900) as compared to saline (~8,000) (Figure 9D). These results indicate that neither cutaneous APC nor draining lymphatics are required for GMCSF-MOG-mediated Treg induction.

### Vaccine-Induced Kinetics Controlling Treg Emergence Was a Function of Pre-existing Treg Levels

A central question was whether tolerogenic GMCSF-MOG vaccination required pre-existing Tregs to stage the rapid and predominant FOXP3^+^ Treg response that occurred in 3–4 days among the circulating MOG-specific repertoire. That is, did GMCSF-MOG drive expansion of pre-existing Tregs or did GMCSF-MOG induce *de novo* differentiation of Tregs from naïve T cell precursors? To gain insight into this question, 2D2-FIG-*Rag1*^−/−^ mice were derived because these mice largely lack FOXP3^+^ Tregs of either thymic or peripheral origin. TCR transgenic *Rag1*^−/−^ naïve T cells however have the capacity to differentiate into iTregs/pTregs (i.e., inducible Tregs, peripheral Tregs) upon TGF-β signaling during cellular activation.

**Figure 10:**
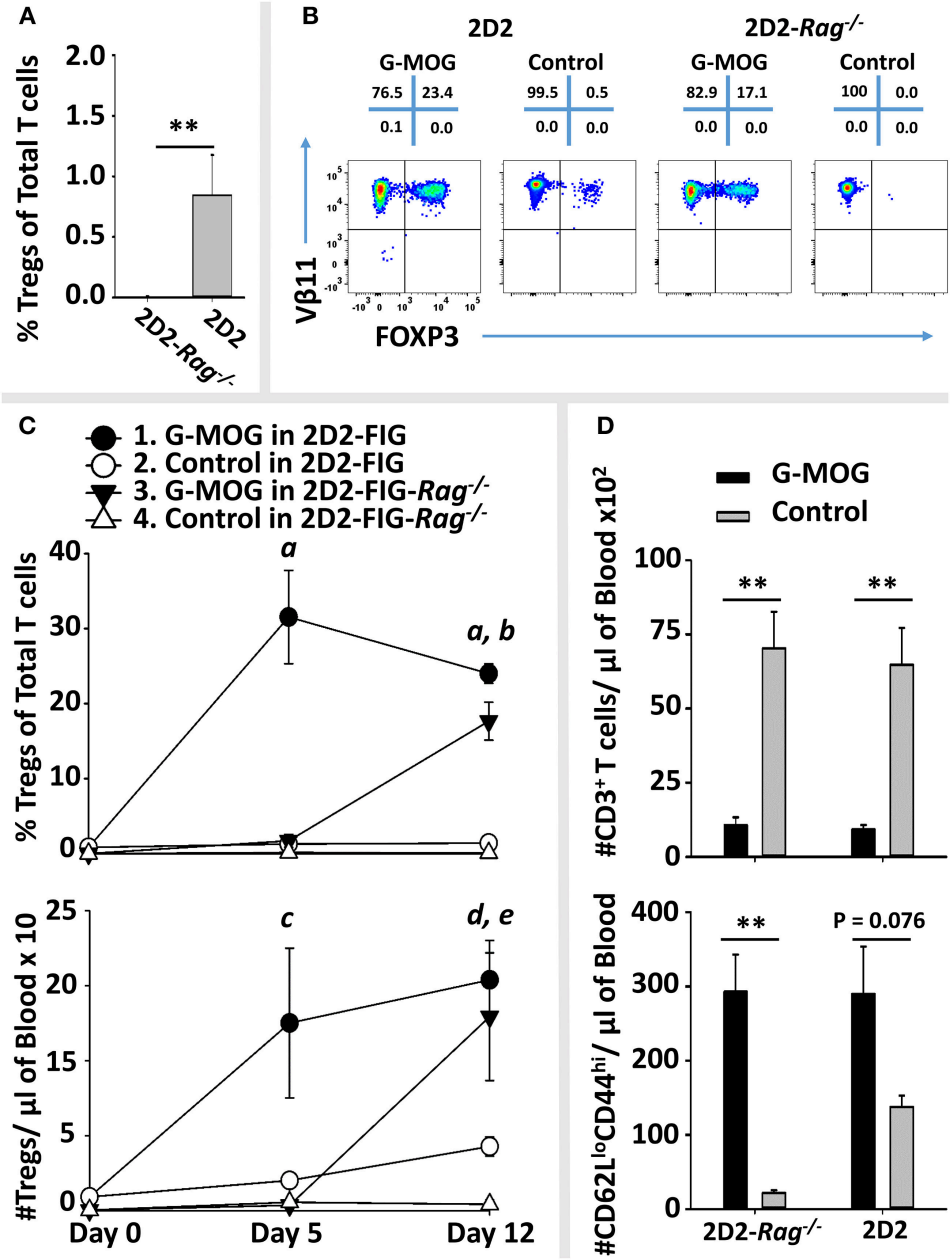
Pre-existing FOXP3^+^ MOG-specific Tregs are associated with rapid expansion of Tregs following GMCSF-MOG (G-MOG) vaccination. **(A)** Shown are percentages of circulating FOXP3^+^ Tregs in the CD3^+^ CD4^+^ T cell pool of naïve untreated 2D2-FIG (*n* = 13) and 2D2-FIG-*Rag1*^−/−^ (*n* = 19) mice. **(B–D)** On day 0, 2D2-FIG (*n* = 5) and 2D2-FIG-*Rag1*^−/−^ (*n* = 4) mice were vaccinated subcutaneously with 4 nmoles of GMCSF-MOG or with control vaccines (saline alone in 2D2-FIG mice or “4 nmoles GM-CSF + 4 nmoles MOG35-55” in 2D2-FIG-*Rag1*^−/−^ mice). PBMCs were analyzed on day 5 and 12. **(B)** Shown are representative dotplots of CD3^+^ CD4^+^ T cells analyzed for FOXP3 expression (x-axis) and Vβ11 (y-axis) on day 12 post vaccination. **(C)** Shown are percentages (top) and numbers (bottom) of FOXP3^+^ Tregs in the CD4^+^ T cell pool on days 5 and 12. Group sizes for day 0 were supplemented with historical data (2D2-FIG mice, total *n* = 50 and *n* = 13; and 2D2-FIG-*Rag1*^−/−^ mice, *n* = 19 and *n* = 10) for calculation of average Treg percentages and Tregs per μl of blood, respectively. **(D)** Shown are the number of CD3^+^ T cells (top) and the number of CD62L^low^ CD44^high^ T cells (bottom) per μl of blood on day 12. Statistical significance was analyzed by use of a one-way ANOVA. Pairwise comparisons were performed by use of the Holm-Sidak method (**p < 0.01). Statistically significant (*p* < 0.05) pairwise comparisons for **(C)**; *a*, 1 vs. 2, 3, and 4; *b*, 3 vs. 2 and 4; *c*, 1 vs. 4; *d*, 1 vs. 2 and 4; *e*, 3 vs. 4. These data are representative of three independent experiments.

As expected, naïve 2D2-FIG-*Rag1*^−/−^ mice exhibited a substantial 120-fold reduction in Treg percentages in that 2D2-FIG-*Rag1*^−/−^ mice averaged 0.007% Tregs compared to 0.845% 2D2-FIG Tregs in the circulating CD4^+^ pool (Figure 10A). These data indicated that 2D2-FIG-*Rag1*^−/−^ mice had profound reductions in Tregs but were not devoid of Tregs. Notably, 2D2-FIG-*Rag1*^−/−^ mice exhibited substantially delayed kinetics in response to tolerogenic GMCSF-MOG, which elicited 1% and 30% Tregs in 2D2-FIG-*Rag1*^−/−^ and 2D2-FIG mice, respectively, by day 5 (Figure 10C, Top). In accordance, 2D2-FIG-Rag1^−/−^ mice and 2D2-FIG mice averaged 4 and 150 Tregs per μl of blood respectively (Figure 10C, bottom) on day 5. By day 12 however, GMCSF-MOG vaccinated 2D2-FIG-*Rag1*^−/−^ mice had high numbers and percentages of circulating Tregs that closely approximated the circulating Treg population in 2D2-FIG mice (~20% Tregs, Figure 10C). Mice injected with saline or “GM-CSF + MOG” control vaccines did not exhibit significant increases in Tregs (Figure 10B). The delayed Treg induction in 2D2-FIG-*Rag1*^−/−^ mice was not due to deficient exposure to MOG35-55 because GMCSF-MOG caused equivalent decrements in circulating Tcon numbers and equivalent elevations in CD44^high^ CD62L^low^ T cell numbers (per μl of blood) in both mouse strains by day 12 (Figure 10D).

Collectively, these data are consistent with the hypothesis that GMCSF-MOG vaccination selectively amplifies pre-existing Tregs to stage the rapid accumulation of Tregs. However, these data did not exclude the possibility that GMCSF-MOG may also drive *de novo* differentiation of naïve T cells into Tregs, perhaps abetted by pre-existing Tregs. Notably, when given sufficient time, GMCSF-MOG has sufficient tolerogenic efficacy to elicit large Treg populations in both 2D2-FIG-*Rag1*^−/−^ and 2D2-FIG mice. These data reveal that the tolerogenic activity of GMCSF-MOG does not require clonotypic diversity in the T cell repertoire or an intact B cell repertoire because 2D2-FIG-*Rag1*^−/−^ mice lack endogenous TCR-alpha chain rearrangements and are largely devoid of B cells.

## Discussion

### The GM-CSF Domain of GMCSF-NAg Mediates Antigen-Targeting and APC-Conditioning to Elicit Tregs

Fusion proteins comprised of GM-CSF and major encephalitogenic peptides of myelin are potent NAg-specific tolerogenic vaccines. When administered before disease onset, these vaccines prevent the subsequent induction of EAE. When administration is initiated after onset of severe paralytic disease, these vaccines are therapeutic interventions that reverse EAE (28–32). This study provides two lines of evidence that GMCSF-NAg tolerogenic vaccines mediate NAg-specific tolerogenic activity, at least in part, by induction of CD25^high^ FOXP3^+^ regulatory T cells (Tregs). First, the prophylactic activity of GMCSF-NAg was reversed by the subsequent treatment with an anti-CD25 mAb that depleted the CD25^high^ Treg subset *in vivo*. GMCSF-MOG vaccinated mice treated with the anti-CD25 PC61 mAb exhibited severe EAE comparable to non-vaccinated littermates (Figure 1). Second, SC GMCSF-NAg administration in saline elicited a rapid Treg response in 2D2-FIG mice that developed in 3 days and persisted as circulating Tregs for several weeks (Figures 2, 4, 5). The vaccine-induced Treg response was robust in that ~20–50% of circulating T cells were FOXP3^high^ and expressed the Vβ11^+^, Vα3.2^+^ 2D2 TCR. This MOG-specific Treg population was also expanded in the spleen and lymph nodes (Figure 3). These data indicated that GMCSF-NAg targeted NAg to a myeloid APC compartment specialized for induction, expansion, and/or maintenance of FOXP3^+^ Tregs.

Several previous studies showed that GMCSF-NAg fusion proteins targeted NAg to myeloid APC for enhanced presentation *in vitro* (32). In the presence of enriched myeloid APC, GMCSF-NAg exhibited antigenic activity that was substantially more potent than NAg alone. For example, the antigenic activity of a rat GMCSF-NAg was 1,000-fold more potent than NAg alone, and this potency enhancement was reversed by free soluble GM-CSF but not by free soluble M-CSF. Likewise, the antigenic potency of a MCSF-NAg fusion protein was blocked by free soluble M-CSF but not by GM-CSF. The enhanced potency of GMCSF-NAg was observed in cultures with purified myeloid APC but was absent in cultures of B cell or T cell APC (32). Murine fusion proteins, including GMCSF-PLP139-151 and GMCSF-MOG35-55 also exhibited an enhancement of antigenic potency due to antigenic targeting of the NAg domain to myeloid APC (30, 31). Antigenic targeting by GMCSF-NAg was also evident in antigen pulse experiments in which GMCSF-MOG was qualitatively more active than “GM-CSF + MOG35-55” in targeting NAg to myeloid APC for the subsequent NAg presentation (29). The GM-CSF domain of GMCSF-NAg not only facilitated antigen targeting for MHCII-mediated NAg presentation, the GM-CSF domain also elicited immunoregulatory activities including an IFN-γ-dependent competence for NAg-stimulated nitric oxide production that abrogated T cell expansion (29). These data indicated that the antigen-targeting and myeloid APC-conditioning activities of GMCSF-NAg may facilitate a mechanism of negative antigen presentation that favors regulatory T cell responses over immunogenic responses.

The physical linkage between the GM-CSF domain and the NAg domain was required not only for targeted antigen presentation *in vitro*, physical linkage was also required for tolerogenic activity *in vivo* as shown in rat and mouse models of EAE. GMCSF-NAg elicited tolerance that suppressed EAE whereas control vaccine formulations including a mixture of “GMCSF + NAg,” GM-CSF alone, NAg alone had no effect (30–32). Covalent GMCSF-NAg linkage was required in both prophylactic and interventional models of EAE. Covalent GMCSF-NAg linkage was also required for induction of a robust Treg response and depletion of the MOG-specific T cell repertoire (Figures 2, 3, 5). The requirement for GMCSF-NAg linkage may reflect a common mechanism of targeted antigen presentation as the basis for different dimensions of the vaccine-mediated response, including tolerance induction, therapeutic intervention, inhibitory antigen presentation, Treg induction, Tcon depletion, and TCR desensitization.

FOXP3^+^ Tregs have been studied extensively over the past several decades, but the “modus operandi” that governs Treg specification and function remains a mystery of contemporary immunology (62). Tregs are maintained in specialized environments characterized by cytokines and antigens that maintain viability, phenotypic stability, and functional activity. The cytokine environment that sustains Tregs is comprised of low-intensity, chronic IL-2 stimulation that favors competitive Treg dominance due to superlative CD25 expression (63). A specialized antigenic niche may also be a necessary foundation for the induction and maintenance of the FOXP3^+^ Treg repertoire (64–70). Although the existence of specialized antigenic compartments for Tregs is commonly assumed by many scientists, the physical and functional basis for such an antigen compartment remains hypothetical. This study supports the concept of an antigenic Treg niche, because a reasonable interpretation is that GMCSF-NAg selectively loads NAg into specialized antigen-processing domain of myeloid APC to confer an antigenic niche that drives dominant NAg-specific FOXP3^+^ Treg responses.

### The NAg Domain of GMCSF-NAg Vaccines Couples Inefficient TCR Recognition With Treg Induction in Both Quiescent and Inflammatory Environments

GMCSF-MOG imposed tolerance in quiescent non-stimulated environments as well as in strongly immunogenic environments, including a CFA-primed lymphatic drainage (29). Thus, GMCSF-NAg and the associated mechanism of inhibitory antigen presentation ameliorated EAE even when GMCSF-MOG or GMCSF-PLP was mixed in the CFA emulsion with the relevant encephalitogenic peptide. This observation contradicts the dogma that APC in quiescent “steady-state” environments are tolerogenic whereas APC in an activated inflammatory environment are immunogenic. Clearly, mechanisms of tolerance must also function in inflamed tissues during adaptive immune responses. Otherwise, immunity to foreign antigens would routinely lead to pathogenic autoimmunity, particularly in response to persistent environmental or infectious agents. Similar difficulties lie in ascribing the Treg niche to either immature vs. mature DC because both types of DC have suppressive properties in select experimental models (71–74). This “DC maturity” paradigm explains the adaptive exposure of peripherally-acquired antigens within secondary lymphoid organs but does not support a conceptually cohesive argument as to why immature vs. mature DC would, respectively, favor regulatory or immunogenic responses (or vice versa). GMCSF-MOG, when emulsified with CFA with a vast excess of encephalitogenic peptide, nonetheless inhibited EAE even though the draining lymphatics are replete with activated, mature DCs. These considerations indicate that the tolerogenic activity of GMCSF-NAg may involve parameters apart from quiescence or immaturity of the myeloid APC subset.

Although the GM-CSF domain was critical for antigen targeting, APC-conditioning, and inhibitory antigen presentation, a central finding of the current study was that the NAg domain was also critical for tolerogenic responses based on the quality of TCR-dependent signaling (Figures 6, 7). Thus, GMCSF-MOG and GMCSF-NFM interact with the same 2D2 transgenic TCR but did or did not exhibit Treg inductive activity in association with low vs. high efficiency TCR interactions, respectively. Likewise, GMCSF-OVA, which contained an epitope recognized as a high-affinity ligand in OTII-FIG mice, lacked Treg inductive activity. These findings indicate that GMCSF-targeting of low-efficiency vs. high efficiency NAg/TCR interactions favors differentiation of regulatory vs. conventional T cell subsets, respectively. One possibility is that inefficient TCR recognition resulted in low levels of CD40L induction on CD4^+^ T cells and consequently inefficient CD40 engagement and low levels of APC-mediated costimulation. Other related non-exclusive possibilities could be also considered. For example, targeted low-affinity TCR interactions may result in limiting levels of IL-2 sufficient to support CD25^high^ Treg responses. Low levels of IL-2 may be insufficient to sustain Tcon cells that express intrinsically lower levels of CD25. Notably, low-affinity NAg may be the common rule because several GMCSF-NAg fusion proteins that incorporated diverse encephalitogenic myelin peptides, including MBP72-86, PLP139-151, and MOG35-55, were potent tolerogens in EAE. The low affinity nature of myelin peptides may reflect generalized mechanisms of self-tolerance, whereby high-affinity self-reactive clones are deleted during maturation and only low-affinity self-reactive clones persist in the periphery. Although high-affinity NFM13-37 peptide may represent an exception in regard to the specific 2D2 clonotype, the overall NFM-reactive repertoire may primarily comprise low-affinity clones because NFM13-37 lacks encephalitogenic activity in C57BL/6 mice (60).

As noted for tolerance induction, the ability of GMCSF-MOG to elicit Treg responses was replete in either quiescent or adjuvant-primed immunogenic environments. That is, GMCSF-MOG emulsified in the Th2 adjuvant Alum or the Th1-adjuvant CFA elicited Treg responses that were equal to that of GMCSF-MOG (in saline) during 14–21 days post-vaccination (Figure 8). Importantly, GMCSF-MOG/Alum or GMCSF-MOG/CFA both exhibited robust Treg-inductive activity, but a mixture of GM-CSF and NAg in CFA lacked Treg inductive capability. Thus, GMCSF-MOG exhibited a requirement for covalent linkage of cytokine and NAg domains when emulsified in CFA adjuvant or when administered in saline. One might assume that the incorporation of unlinked GM-CSF and NAg in an adjuvant would provide a non-covalent physical association in that both entities were physically sequestered in the same antigen depot, but this type of physical association was not sufficient to support the induction of Tregs. These data provide evidence that GMCSF-MOG must retain physically-connected cytokine and NAg domains after immunological extraction from the emulsion during subsequent and perhaps indirect antigen processing steps that lead to tolerogenic antigen presentation.

### GM-CSF Has Complex Proinflammatory and Anti-inflammatory Activities

A substantial literature supports the concept that GM-CSF has a pro-inflammatory role in immunity, including autoimmunity and EAE. For example, GM-CSF-deficient mice exhibited profound resistance to EAE, and anti-GM-CSF mAb inhibited EAE (75–81). However, treatment of GM-CSF-deficient mice with an anti-CD25 mAb that depleted CD25^high^ FOXP3^+^ Tregs restored full disease susceptibility in both active and passive models of EAE (58). These data indicate that the Csf2^−/−^ mice lack the responsive capability to overcome the natural resistance of the Treg repertoire in this model of EAE. A substantial literature also supports the concept that GM-CSF has a profound tolerogenic role in immunological disease. Administration of exogenous GM-CSF is known to inhibit several models of autoimmunity, including experimental autoimmune myasthenia gravis, experimental autoimmune thyroiditis, and type I diabetes via the induction of “tolerogenic DC” and regulatory T cell subsets (43, 46–56, 82–91). This study may provide insight into immunogenic vs. tolerogenic roles of GM-CSF in that GM-CSF-conditioned myeloid DC may integrate the intrinsic efficacy of TCR signaling within a local environment or across an immunological synapse to establish dominance of conventional or regulatory T cell responses.

### The GMCSF-NAg Vaccines Have Unique Tolerogenic Activities

The observation that GMCSF-NAg, when emulsified in CFA, retains both Treg-inductive and tolerogenic activities reveals unique attributes of this tolerogenic vaccine platform. Many other types of tolerogenic vaccine, if emulsified in a pro-inflammatory adjuvant like CFA, would likely cause rather than prevent autoimmune pathogenesis. Another unique attribute of the GMCSF-NAg vaccine platform is that both SC and IV routes were equally effective for induction of robust Treg responses (Figure 9). Other vaccine platforms are contingent upon intravenous administration whereas SC administration is countermanded and potentially immunogenic. One assumes that SC administration of GMCSF-MOG introduces the vaccine in the lymphatic drainage to stage the tolerogenic response. However, the strong tolerogenic efficacy of intravenous GMCSF-MOG indicates that blood-borne immunological organs (spleen, liver, etc.) are sufficient to drive Treg responses without need for draining lymphatics.

### The Expansive vs. Inductive Roles of GMCSF-MOG in Driving Emergence of MOG-Specific Tregs

At least two mechanisms may account for the differential kinetics of the vaccine-induced Treg response in 2D2-FIG-*Rag1*^−/−^ vs. 2D2-FIG mice. First, GMCSF-MOG may expand pre-existing Tregs and may lack efficacy for *de novo* induction of Tregs from naïve precursors. In this model, GMCSF-MOG vaccination of 2D2-FIG-*Rag1*^−/−^ mice may drive Treg expansion from the minute population of pre-existing Tregs, which would require a prolonged 10–12 day duration to reach peak accumulation. Second, GMCSF-MOG may induce the *de novo* differentiation of iTregs by mechanisms facilitated by pre-existing Tregs. In this case, Treg induction would be slower in 2D2-FIG-*Rag1*^−/−^ mice while pre-existing Tregs slowly facilitated expansion of peripherally-induced Treg populations by a mechanism of infectious tolerance. These models are not exclusive, and aspects of both models may drive the tolerogenic Treg response. Alternatively, the differential kinetics of the vaccine-induced Treg response in 2D2-FIG-*Rag1*^−/−^ vs. 2D2-FIG mice may reflect other unappreciated differences between the two strains apart from the pre-existing Treg levels. Future research will be needed to resolve these possibilities.

## Conclusion

GMCSF-NAg fusion proteins comprise a unique tolerogenic vaccine platform. The emerging picture is that the GM-CSF domain interacts with the GM-CSF receptor (CD116, CD131) on myeloid APC to target the NAg domain to myeloid APC contingent upon the covalent GMCSF-antigen linkage. The subsequent GM-CSF/receptor interaction conditions myeloid APC to express co-inhibitory activities that facilitate an overall process of inhibitory antigen presentation. Presentation of NAg domain as an inefficient TCR ligand to myelin-reactive CD4^+^ T cells drives desensitization of the respective myelin-reactive T cell repertoire and promotes the induction and outgrowth of myelin-specific CD25^high^ FOXP3^+^ Tregs. The overall vaccine response mediates active dominant tolerance in the context of inflammatory environments. This tolerogenic vaccine platform can provide mechanistic insight into basic Treg physiology while advancing a unique vaccine class to fulfill an unmet clinical need in MS.

## Author Contributions

CM, AC, AB, and MM designed the project, provided intellectual input, analyzed the data, and wrote the manuscript. CM performed most of the experiments. AC was instrumental in the initiation of the project. AB and SE contributed to important aspects of the experimentation.

### Conflict of Interest Statement

The authors declare that the research was conducted in the absence of any commercial or financial relationships that could be construed as a potential conflict of interest. Animal care and use was performed in accordance with approved animal use protocols and guidelines of the East Carolina University Institutional Animal Care and Use Committee.

## Abbreviations

APC: antigen presenting cell(s)
CFA: Complete Freund's Adjuvant
CNS: central nervous system
EAE: experimental autoimmune encephalomyelitis
FIG: Foxp3-IRES-GFP knock-in allele
GM-CSF: granulocyte-macrophage colony stimulating factor
IV: intravenous
M-CSF: macrophage colony stimulating factor
MFI: mean fluorescence intensity
mAb: monoclonal antibody
MS: multiple sclerosis
MBP: myelin basic protein
MOG: myelin oligodendrocyte glycoprotein
NAg: neuroantigen(s)
NFM: neurofilament medium protein
OVA: ovalbumin
PBMC: peripheral blood mononuclear cells
PLP: proteolipid protein
SC: subcutaneous
SE: Standard error of the mean
Tcon: conventional T cell(s)
TCR: T cell antigen receptor
Treg: regulatory T cell(s).
